# Depression of the Melting Point in Naturally Grown Circular Crystals

**DOI:** 10.1021/jacsau.5c01455

**Published:** 2026-02-06

**Authors:** Shengzhe Jia, Xin Su, Yonghui Wang, Jiaqiang Liu, Ejaz Ahmed, Liang Li, Weiwei Tang, Panče Naumov, Xiaoyan Cui, Junbo Gong

**Affiliations:** † School of Chemical Engineering and Technology, State Key Laboratory of Chemical Engineering and Low-Carbon Technology, The Co-Innovation Center of Chemistry and Chemical Engineering of Tianjin, 12605 Tianjin University, Tianjin 300072, People’s Republic of China; ‡ Department of Chemistry, School of Chemistry and Molecular Engineering, 12655 East China Normal University, 500 Dongchuan Road, Shanghai 200241, People’s Republic of China; § College of Chemistry, 12538 Nankai University, Tianjin 300071, People’s Republic of China; ∥ Smart Materials Lab, 167632 New York University Abu Dhabi, P.O. Box 129188, Abu Dhabi, UAE; ⊥ SAFIR Novel Materials Development Lab, Sorbonne University Abu Dhabi, P.O. Box 38044, Abu Dhabi, UAE; # Center for Smart Engineering Materials, New York University Abu Dhabi, P.O. Box 129188, Abu Dhabi, UAE; ∇ Research Center for Environment and Materials, Macedonian Academy of Sciences and Arts, Bul. Krste Misirkov 2, MK-1000 Skopje, Macedonia; ○ Department of Chemistry, 5894 New York University, 100 Washington Square East, New York, New York 10003, United States

**Keywords:** polymorphism, ROY, organic crystal, melting point, mechanical properties

## Abstract

The melting point is one of the most fundamental properties of crystalline materials and has been commonly used to determine the chemical and phase purity of organic compounds. Here, we report a significant depression in the melting point of about 1.4 K in naturally grown crystals with a circular shape obtained for one polymorph of the tetradecamorphic compound 5-methyl-2-((2-nitrophenyl)-amino)-3-thiophenecarbonitrile (ROY). Crystals of ROY grown by microspace sublimation have peculiar habits, with irregular, elongated, occasionally bent, or curled habits, and with one or both of their ends closed into loops. In contrast to the straight crystals, the Mueller matrix microscopic analysis suggested the continuous reorientation of electrical dipole moments in the curled crystals. When heated, these curly crystals often start to melt at the kink and exhibit a lower melting onset point and a broader endothermic peak in the thermal (DSC) fingerprint compared with the regular crystals. The decrease in the melting point was found to be proportional to the deformation expressed as the curvature of the crystals; it is also inversely proportional to the crystal width for narrow crystals, but it is independent of the width for wider crystals. The bent section of the crystals is mechanically softer than the straight part, and both the stiffness and hardness are inversely proportional to the degree of curling, presumably due to defects. In the three-dimensional reciprocal space, the curled crystals show diffuse diffraction or streaks due to lattice distortion. Nanoinfrared spectroscopic signatures indicate that the lattice distortion is related to the conformational changes of the molecule. The results highlight the dependence of the extent of crystal deformation on melting properties, which may have broad implications for modulating properties of pharmaceutical crystals.

## Graphical abstract

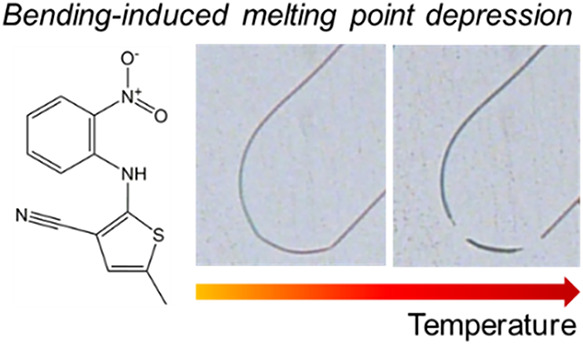

## Introduction

The relationships between the size, shape, and properties of organic crystals hold the key to their properties that are central to some of the main industrial sectors, including production and utility of fine chemicals, pharmaceuticals, fibrous materials, and high-energy materials (explosives).^1–4^ The apparent softness, brittleness, and proneness to abrasion of organic crystals have long stood as two major impediments to the expansion of the scope of application of these materials to emerging fields, such as flexible electronics, soft robotics, and organic optics. However, the discovery of the flexibility,^5–8^ thermo/photo/mechanosalient (jumping),^9,10^ self-healing,^11–13^ and shape-memory^14,15^ effects of some organic crystals has brought a paradigm shift in the solid-state chemistry research, that extends academic curiosity and could have far-reaching implications for organic optics, memories, sensors, and electronics.^16–27^ When they are subjected to forces in appropriate directions, many slender, lightweight, flexible organic crystalline materials can be temporarily or permanently bent into arcs with an arbitrary curvature, twisted into helices, and even curled into loops.^28–31^ These captivating macroscopic deformations cause perturbations in their crystal structure and consequently and inevitably also alter their physical properties. In basic mechanical tests, simple manual operations such as pressing a crystal against a solid, harder object(s) are often used to induce deformation, and these manipulations can be performed even on microcrystals by using controlled mechanical probes or microcantilevers.^5,32–35^ However, such processes are labor-intensive, require automation for precise control, and oftentimes introduce defects, fractures, and other artifacts that are not inherent to the original crystal deformation. While some crystals can be deformed by external intervention, others tend to naturally grow in nontrivial shapes during crystallization.^36–48^ Unlike manual bending of individual crystals, such bending, twisting, or curling during crystal growth comes with the added potential of scalability, provided that robust crystallization methods are established.^22,49^ Crystal deformation can also be accomplished by using light,^50,51^ infrared radiation,^52^ humidity,^53,54^ magnetic field,^55^ low temperature,^21,56^ and most of these can be also conveniently realized with heterogeneous structures, such as hybrid crystal–polymer composites.^57^

One of the immediately relevant physical properties that depends on the crystal structure and also stands as an essential characteristic of any organic crystal is its melting point, the temperature at which the solid and liquid phases of the pure compound have identical Gibbs energies. It reflects the thermodynamic stability of the lattice of a perfect crystal, and its value determined in practice depends on the crystal size, conformation, molecular arrangement, and chemical purity,^58,59^ among other experimental factors. Since the crystallographic practice usually requires well-defined, defectless, and regular crystals, the effects of deformation on the melting have received less attention^60,61^ relative to some other contributing factors. Several studies have concluded that, for instance, the melting point and entropy of chlorobenzene, heptane, and benzene nanocrystals are size-dependent.^62–64^ A more recent study^65^ discovered a decrease in the melting point of plastically bent crystals, which was attributed to increased contribution from defects. However, quantitative relationships between the crystal deformation and the melting of naturally deformed crystals or crystals where the curvature is systematically varied are not available yet. Mechanical bending of straight crystals inevitably causes defects, and this process is to any practical degree completely stochastic. Here, we report naturally grown deformed crystals of one of the forms of the very well-known polymorphic compound 5-methyl-2-[(2-nitrophenyl)­amino]­thiophene-3-carbonitrile (ROY, Figure 1a), a material which, due to its extraordinarily rich polymorphic landscape, is often used for both experimental and computational studies into factors that affect small-molecule polymorphism. ROY is also a popular material due to its extensive polymorphism, with 14 forms identified to date.^66^ We recently observed that when subject to the microspacing sublimation technique,^67^ the compound deposits as crystals with peculiar shapes. In the course of their physical characterization, these crystals were found to have significantly lower melting points than the other regular crystals of the same form, and here, we investigate the dependence of the melting on the degree of deformation. This report details these observations and provides a possible explanation for their occurrence, particularly with regard to intricacies related to the molecular conformation.

**Figure 1:**
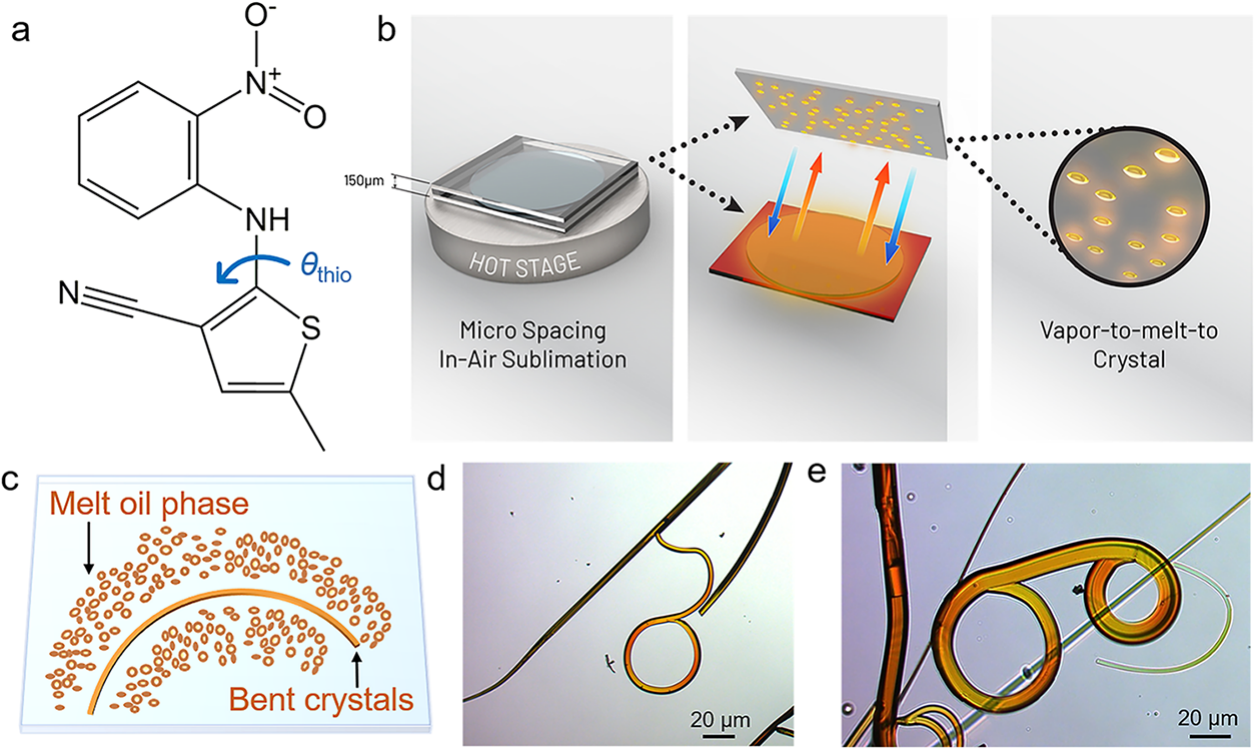
Chemical structure and preparation of ROY crystals with unusual shape by microspacing sublimation. (a) Molecular structure of ROY showing the torsion angles θ_thio_ that are usually used as characteristic geometric parameters to describe the molecular conformation. (b) Schematic of the microspacing sublimation used to grow the deformed crystals. (c) Proposed mechanism for the growth of bent crystals through an intermediate oil phase. (d, e) Polarized optical images of typical (d) bent and (e) circular crystals.

## Results and Discussion

### Preparation of Naturally Curled Crystals

The crystal structures of 12 polymorphs (the abbreviations used in the related literature are Y, ON, R, YN, OP, ORP, YT04, Y04, R05, PO13, R18, and Y19) out of 14 known solid forms of ROY have been determined by single-crystal X-ray diffraction.^66^ The torsion angle θ_thio_, which represents the angle between the C–N and C–S bonds (Figure 1a), is believed to be associated with the vivid and distinct color variations among the polymorphs.^68^ When inspected visually, some polymorphs have similar and even nearly identical colors and some forms crystallize under identical conditions, especially when they are prepared from solution. Concomitant crystallization,^69,70^ as well as occasional cross-nucleation,^71,72^ makes the preparation of the target polymorph exceedingly arduous. Preparation of a single form by crystallization from solution or melt without additives or seeding is equally challenging. In this work, by using the microspacing sublimation and deposition technique, we observed the formation of crystals of the orange polymorph (abbreviated as ON) with unusual bent or even curled morphology (Figure 1b). Powder X-ray diffraction of the deformed crystals confirmed that despite the strikingly different shape, they were from the same phase (ON) as the straight crystals (Figures S1 and S2). Growth of bent crystals with irregular morphologies is known to be prominent when nonclassical vapor-to-melt deposition is used, which includes the molten phase as an intermediate to the crystalline one (Figures 1c and S3).^73^ Most crystals grown on the glass substrate were bent, and some longer crystals had one or both of their ends curled into loops, while their middle part was straight (Figure 1d,e). Long acicular crystals were also observed in the vicinity of the bent crystals. In the cross-polarized image of the circular crystal, a bright, birefringent loop with distinct extinction (Maltese cross) was observed at horizontal and vertical orientations parallel to the polarizer and analyzer directions (Figure S4).

The straight crystals of the ON polymorph are in the monoclinic centrosymmetric space group *P*2_1_/*c* with unit cell parameters *a* = 3.85520(10) Å, *b* = 18.4685(4) Å, *c* = 16.3436(3) Å, and β = 92.6(1)° (Table S1). We note that the crystal structure of the ON form has been reported previously. The unit cell parameters and crystal structure obtained in this work are in excellent agreement with those reported previously.^68,74–76^ The as-grown crystals have two prominent pairs of faces, (01̅1̅) and (01̅1), while the smaller face is (1̅00) (Figure 2a). The bent crystals have identical faces to those of the straight crystals; however, the relative surface area of their crystal faces is slightly different (Figure 2b); the bent crystals are often thicker than the straight ones. The crystals obtained by deposition have a needle-like morphology, with their longest axis corresponding to the (1̅00) direction (Figure 2), and thus they naturally tend to lie flat on the substrate. Since no preparation was applied to the sample before the PXRD analysis, the powder diffraction pattern exhibits preferential alignment of the (01̅1̅) planes, resulting in a higher intensity of the corresponding diffraction peaks (Figure S5). We have compared the crystal structure data obtained from powder XRD refinement to those from single-crystal analysis (Table S2), revealing nearly identical unit cell parameters. The intermolecular interactions in the structure of the ON crystal viewed down the (01̅1), (01̅1̅), and (1̅00) faces are illustrated in Figure 2e. The molecules form columns by π-stacking in the direction of the crystallographic *a* axis, which is the fastest growth direction. When viewed down the (1̅00) face, the molecules are interconnected by weak hydrogen bonds, C–H···O (*D*, *d*, θ: 3.483 Å, 2.592 Å, 156.4°; 3.530 Å, 2.655 Å, 153.3°) and C–H···N (3.383 Å, 2.574 Å, 143.2°), which are spread out toward the *b* and *c* axes (Figure S6). The straight ON crystals of millimeter size were found to be mechanically elastic and can be bent to an elastic limit of up to about 1.31% on the (01̅1) face and up to about 0.96% on the (01̅1̅) face (Figure 2c,d).

**Figure 2:**
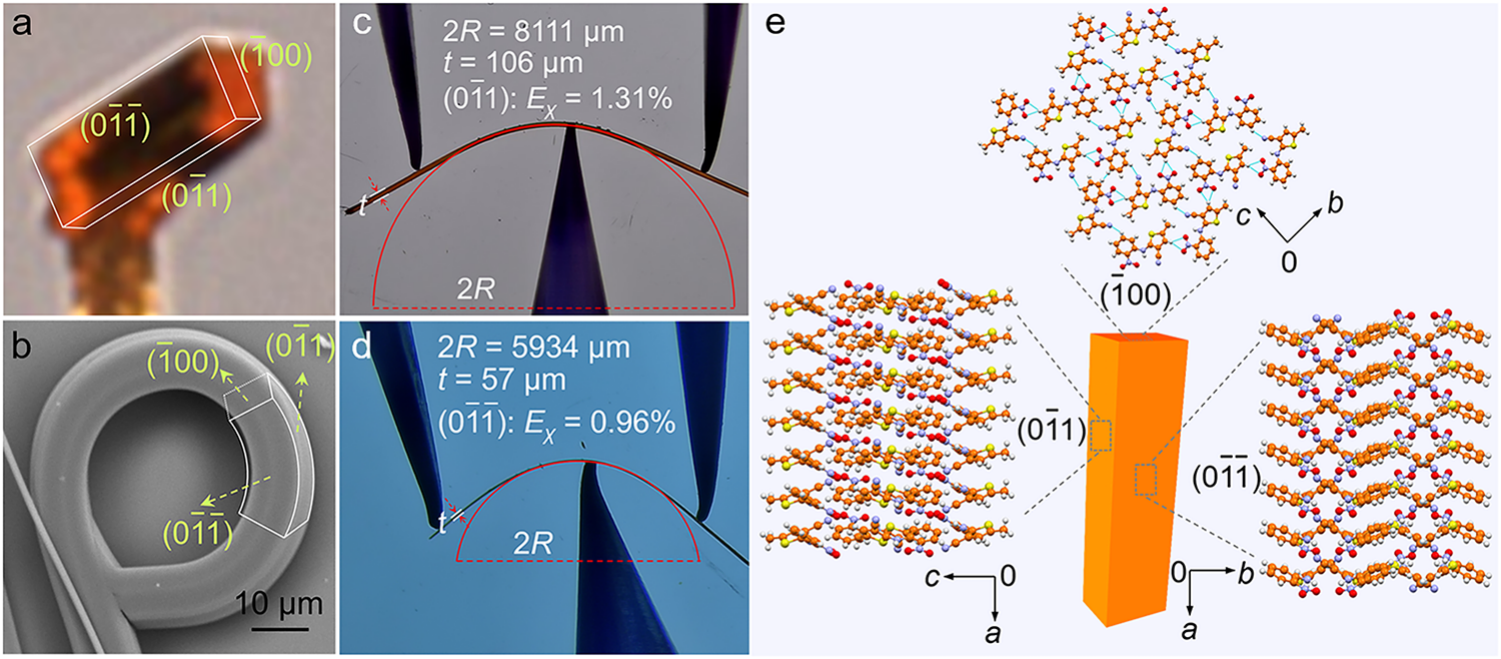
Structure and morphology of the form of ON crystals of ROY. (a) Optical nonpolarized image of a straight crystal. (b) SEM image of a curled crystal. (c, d) Elastic deformation of a crystal of ROY on the (01̅1) crystal face (c) and the (01̅1̅) crystal face (d). (e) Molecular packing structures viewed down the (1̅00), (01̅1), and (01̅1̅) faces.

A custom-built Mueller matrix microscope (MMM) was employed to study the optical properties of crystals of ROY having distinct morphologies (Figures S7 and S8). The structural anisotropy of the crystals governs the refractive index anisotropy, yielding strong linear birefringence (LB) with a specific orientation (LB angle). Similarly, the anisotropic arrangement of the highly absorbing ROY molecules in the crystals results in aligned electric transition dipole moments, leading to the orientation of linear dichroism (LD and LD angle). For the straight crystals, the consistent orientation of the crystals keeps the LB angle and LD angle along the whole crystal (Figure 3a–c). On the contrary, in the naturally grown curled ROY crystals, the LD angle undergoes continuous modulation across a full 360° range as the orientation of ROY crystals varies progressively (Figure 3d–f).^77^

**Figure 3:**
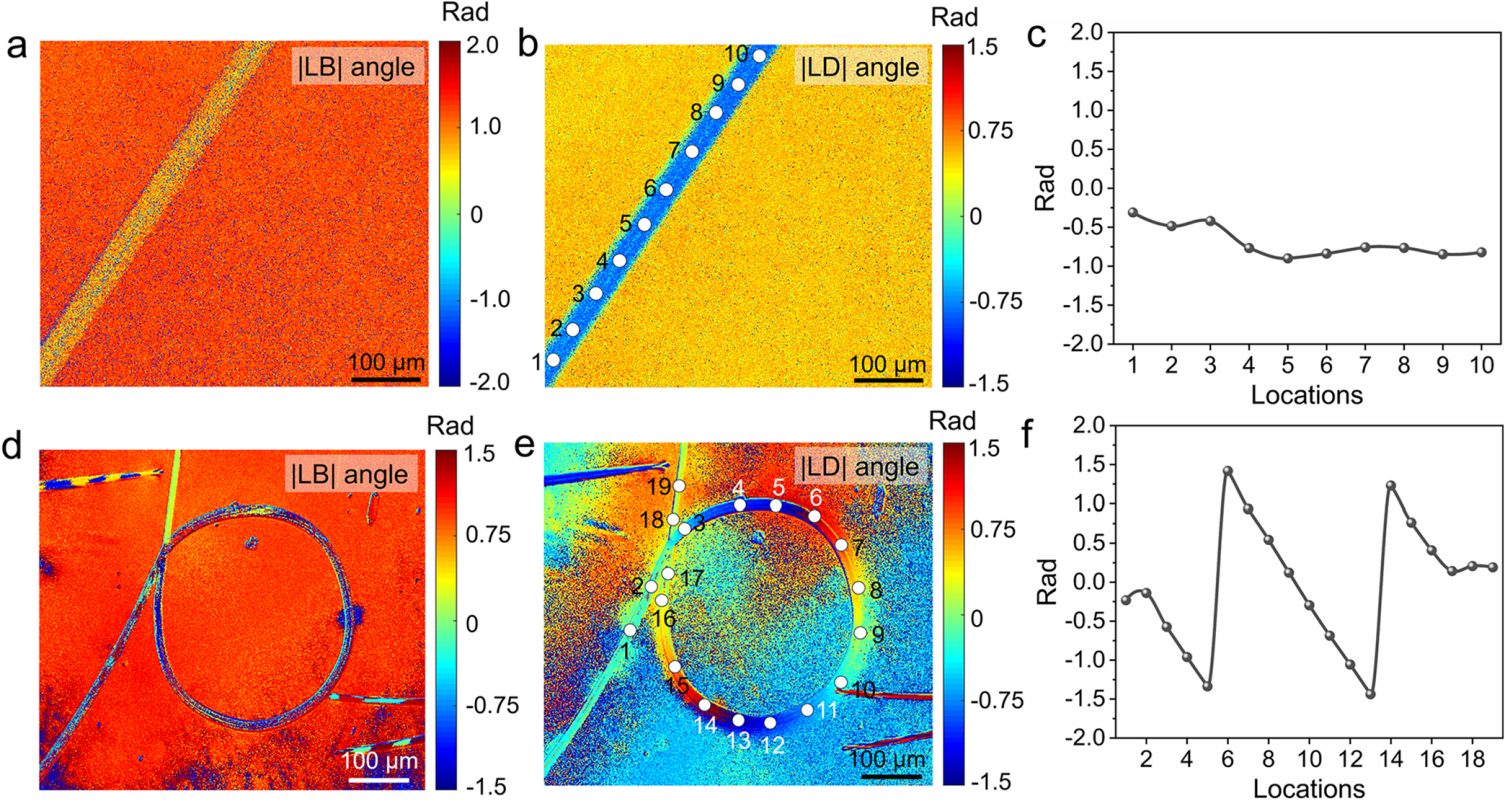
Mueller matrix analysis of straight and curled ROY crystals. (a) |LB| angle mapping of a straight crystal. (b) |LD| angle mapping of a straight crystal. (c) Spatial variation of the |LD| angle at the locations labeled with numbers 1–10 on the straight crystal shown in panel (b). (d) |LB| angle mapping of a curled ROY crystal. (e) |LD| angle mapping of a curled crystal. (f) Spatial variation of the |LD| angle at the locations labeled with numbers 1–19 on the curled crystal shown in panel (e).

### Deformation-Induced Depression of the Melting Point

The thermal behavior of the deformed ROY crystals was observed by heating the crystals on an inverted optical microscope at a rate of 5 K min^–1^. The heated crystals start to melt at the bent segment, and the melting continues in the straight sections (Figure 4a). In differential scanning calorimetry (DSC), this process is accompanied by an endothermic effect around 389 K, which for the bent crystals is composed of more than one peak (Figure 4b). This appearance is different from that of the single sharper peak observed for straight crystals at around 390 K (Figure 4c). Statistical analysis of the melting points revealed that, indeed, the average melting point of the deformed crystals, *T*_m_ = 388.90 ± 0.60 K (*n* = 8 crystals) is about 1.4 K lower than that of the straight ones, at *T*_m_ = 390.31 ± 0.32 K (*n* = 8 crystals; Figure 4e). The thermal parameters of bent and straight crystals are listed in Tables S3 and S4, and the difference in the thermal properties of the bent and straight crystals is statistically significant (Table S5). As can be inferred from the half-peak width (Δ*T*_m_) in the DSC curves, the melting point of the bent crystals exhibits more prominent fluctuations (Figure S9). The half-peak width of the bent crystals was Δ*T*_m_ = 1.61 ± 0.44 K (*n* = 8 crystals), which is significantly larger than that of the straight crystals, Δ*T*_m_ = 1.01 ± 0.19 K (*n* = 8 crystals, Figure 4f). The broadening and low-temperature shift of the endothermic peak are further illustrated in Figure 4d, which shows a plot ofthe half-peak width against the melting point. Similar, yet smaller melting point depression of about 0.4 K and clear peak splitting were previously reported for mechanically bent plastic crystals.^65^ However, in the case of the deformed crystal obtained by crystallization reported here, the peak splitting is less pronounced, perhaps due to the higher randomness of the lattice defects. Previously, Naumov et al.^65^ have developed a phenomenological model to describe the profiles of the DSC peaks of bent and straight crystals. This hybrid model accounts for the statistical character of the bent crystal structure and fundamental thermodynamic principles governing the melting process and can explain the peak broadening and splitting observed with bent crystals. Increased width of the multivariate probability distribution function results in broader peaks. Decreasing the dispersion results in splitting of the DSC peaks. While this model is readily able to explain the appearance of the DSC profiles of straight and bent crystals, given the quasistructural continuity of bent crystals, separating the melting points of the bent and straight regions of the curved crystals is challenging.

**Figure 4:**
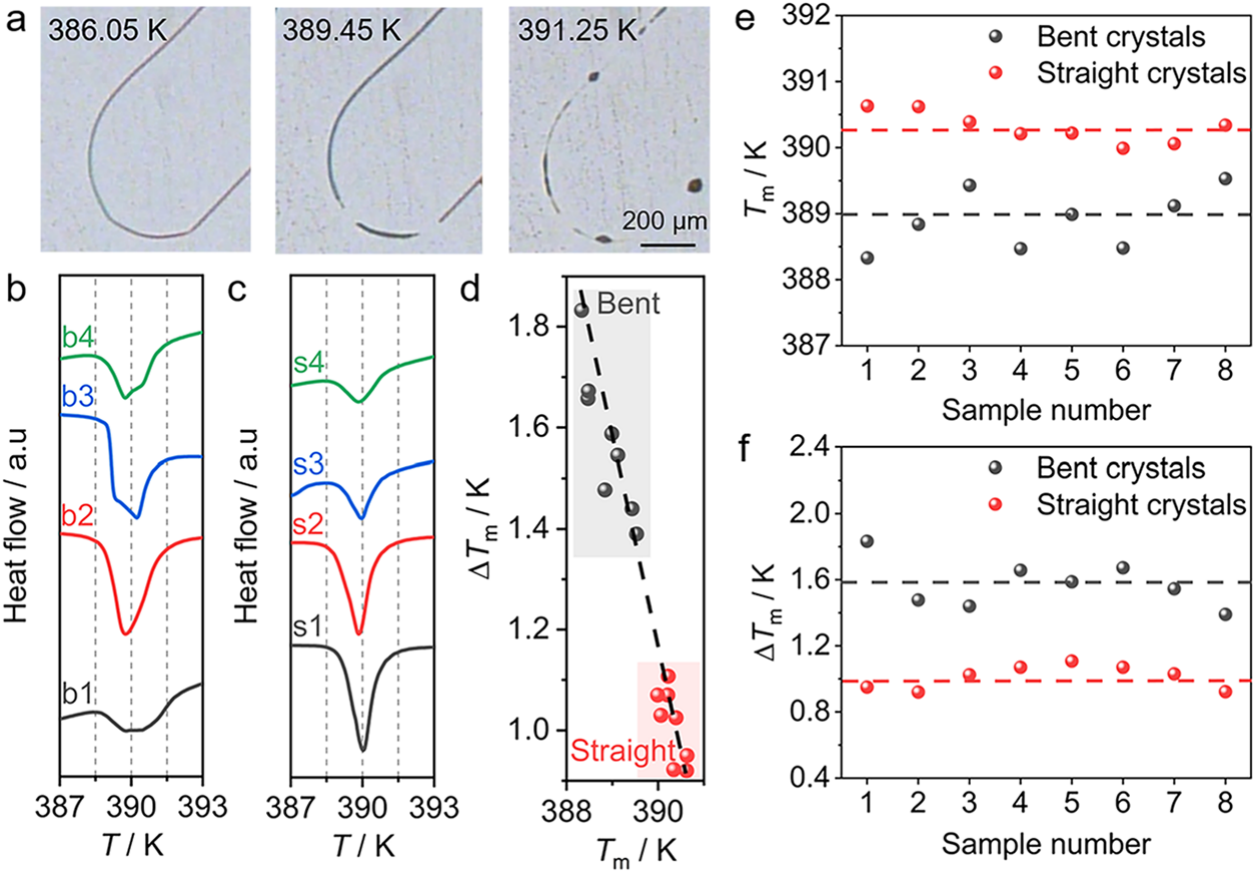
Effect of deformation on the melting point of the ROY crystals. (a) Micrographs of crystals recorded upon heating from 303.15 to 393.15 K at a rate of 5 K min^–1^. (b, c) DSC curves of (b) four bent crystals (labeled b1–b4) and (c) four straight crystals (labeled s1–s4). (d) Correlation of the half-peak width Δ*T*_m_ of bent and straight crystals with the corresponding melting point. (e, f) Variation of the melting point (e) and half-peak width (f) across eight bent and eight straight crystals. The dotted horizontal lines are guides to the eyes to facilitate the inspection of the variations.

We further aimed to decipher the effect of bending curvature (Figure S10) on the extent of the melting point depression. Such analysis is challenging as it requires a substantial amount of data. However, unlike manual bending of crystals, where a large number of samples can, at least in principle, be deformed to an arbitrary curvature, analysis of crystals bent during growth relies on the availability of a sufficient number of such naturally deformed crystals. To that end, we selected and analyzed a total of 84 crystals, and Figure 5 presents the variation of the melting points with their bending curvature and crystal width. Since a deformed crystal has approximately uniform width across its length but may have different bent sectors, the lowest temperature of melting and the corresponding curvature were plotted. Based on their width (*d*), the crystals were batched into seven categories: 1–2 μm, 2–2.5 μm, 2.5–3 μm, 3–4 μm, 4–6 μm, 6–8 μm, and 8–16 μm (Figures 5c and S11 and Table S6). We observed that thinner crystals tend to bend more significantly (Figure 5b); specifically, the bending curvature decreases dramatically for widths *d* = 2–3 μm but declines slowly for crystals that are 3–8 μm wide. Crystals that are wider (*d* = 8–16 μm) usually have smaller curvature.

**Figure 5:**
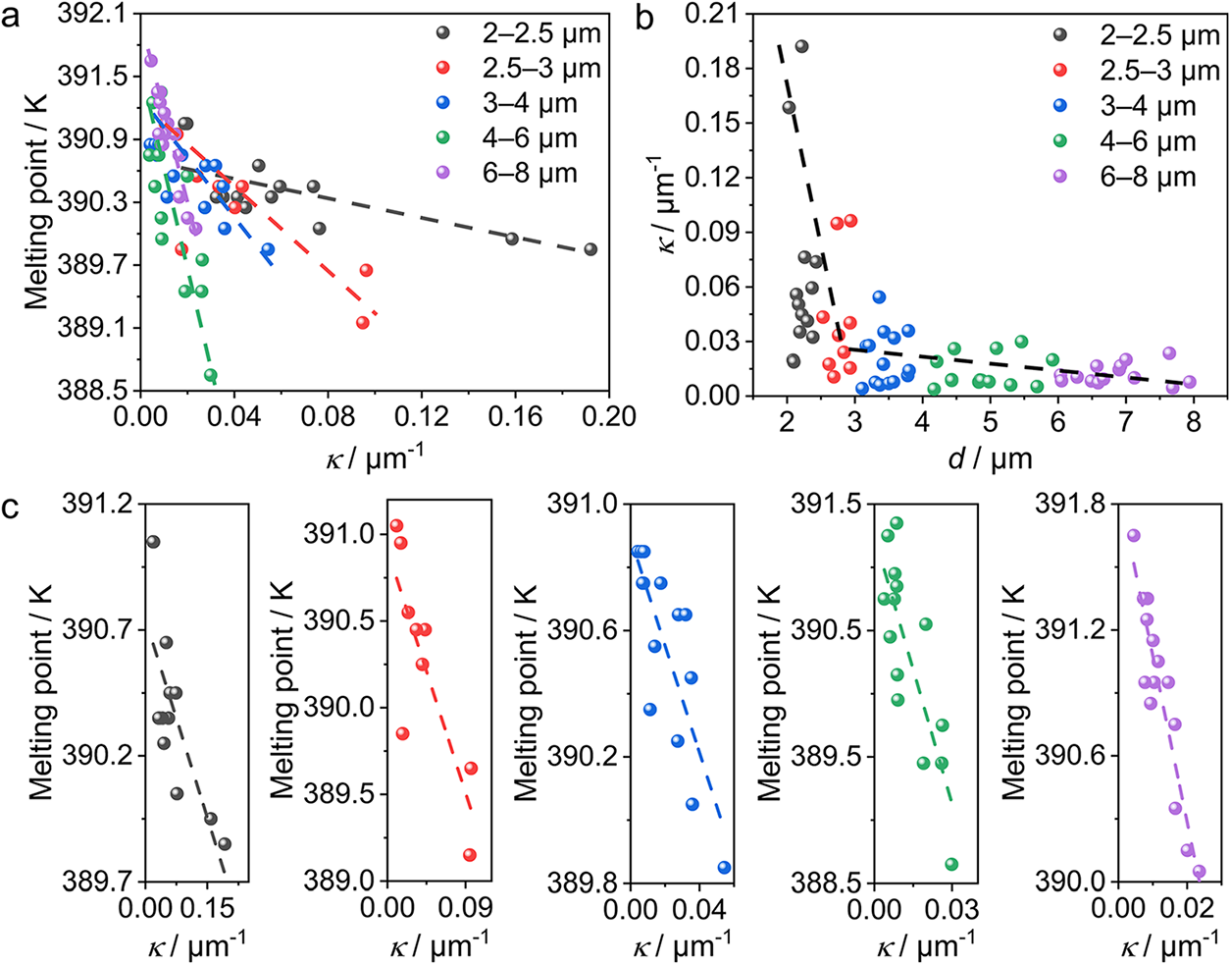
Effects of the bending curvature and width of deformed crystals on their melting point. (a) Correlation of the melting point with the bending curvature, κ. (b) Correlation of the curvature κ with the crystal width *d*. (c) Categorization of the melting point with the curvature κ based on the crystal width (from left to right: 2–2.5 μm, 2.5–3 μm, 3–4 μm, 4–6 μm, 6–8 μm). The dotted lines are the linear fitting results.

Figure 5a shows the melting point against the curvature, and the trends for five width categories are plotted in Figures 5c and S11. Most of the naturally bent crystals of ROY had curvatures below 0.04 μm^–1^ (Figure S12). In contrast, the curvature of the manually induced bending crystals can be manipulated to an arbitrary degree under the appropriate external force. Plastically bendable crystals have been reported previously, including crystals of 4-dibromobenzene^65^ and hexachlorobenzene^78^ with an arbitrary shape, and of 2-hydroxy-5-methoxyacetophenone^79^ with a curvature of 0.06 μm^–1^.

We infer from these plots that in general the melting point decreases with increasing curvature, and this conclusion is consistent with the observation of the temperature of the first observation of the melting of the bent segments (Figure 4a). This behavior may be associated with the defect density in the bent crystals and is consistent with the earlier conclusions^65^ that a bent crystal has a higher Gibbs energy compared with the straight one, resulting in a lower melting point. We suggest that these defects are predominantly dislocations, implied by the defects observed on the surface of bent crystals (Figure S13).^80^ The accumulation of defects could explain the observation that melting is initiated at the kink of the bent crystals.

Further analysis revealed the curvature dependence of the melting point, which shows a nearly linear decrease with the increasing curvature (Figure 5c). This correlation appears to be better for widths *d* = 6–8 μm, and the observation could be explained by the Gibbs–Thomson equation:^65^

1 $$T_{m}=T_{m,\mathrm{bulk}}-\alpha \times \kappa$$

where *T*_m_ is the melting point of a curved solid surface with bending curvature κ, *T*_m,bulk_ is the melting point of a “bulk” crystal (κ = 0), and α represents a material-characteristic constant. The melting point of the curved crystals shows a negative linear correlation with the curvature for different ranges of the crystal width (Figure 5c and Table S7). When the curvature κ = 0, the melting points of the straight crystals are 390.74*–*391.87 K, while the material-characteristic constant α fluctuates over a wide range. The mathematical formalism of the Gibbs–Thomson equation has been established for spherical solid particles, and the exclusion of defects that are inevitably present in the curved crystals precludes us from establishing quantitative correlations beyond its usefulness in reaching qualitative conclusions.

For a single deformed crystal, Nye correlated the bending curvature κ with the number of excess dislocations *n*, presented as κ = *n* × *b*, where *b* is the Burgers vector.^81^ Although this model might not be strictly appropriate for quantitative analysis of the crystal defects at different bending degrees, it suggests that the defect density increases with increasing bending curvature and thus lowers the melting point. At two extreme width categories (i.e., *d* = 1–2 μm and *d* = 8–16 μm), this inverse linear dependency is less significant. When the crystal width is within 1–2 μm, naturally bent crystals are often accompanied by higher defect density, leading to large fluctuations in thermal properties. Wider crystals (*d* = 8–16 μm) are less bent, sometimes deviate from a straight shape only slightly, and correspondingly have higher melting points. We hypothesize that these defects are predominantly dislocations, based on the changes observed on the surface of bent crystals, although a more in-depth study is necessary to characterize their nature. The increased defect concentration could also explain the observation that the melting is initiated at the kink of the bent crystals. Previous studies on the melting of inorganic nanowires, for example, have shown that the melting depends on the nanowire size, and that smaller nanowires tend to have lower defect density and hence a higher melting point.^82,83^ To the best of our knowledge, this is the first observation of linear deformation-induced melting point depression with increasing bending curvature.

In a preceding report, Naumov et al.^65^ have demonstrated the depression of the melting point of a manually deformed plastic organic crystal, 1,4-dibromobenzene. These thermomechanical effects become more pronounced with heavier mechanical damage due to an increased concentration of defects and ultimately result in a large temperature spread of the associated phase change. However, control over the curvature during manual bending of plastic crystals poses practical challenges due to the apparent defects that evolve at the acutely deformed sections. Our future studies will aim to compare the generality of these observations by comparison with other plastically bendable crystals.

### Mechanical Softening of Naturally Bent Crystals

The accumulation of defects in naturally bent crystals is expected to affect the mechanical properties, such as stiffness and hardness, in the bent regions. Therefore, the mechanical properties of three classes of ROY crystals, categorized as straight, slightly bent, and acutely bent based on their extent of deformation (i.e., average curvature), were studied by nanoindentation. Figure 6c–h depicts the force–displacement curves, Young’s modulus (*E*), and hardness (*H*) measured in fixed-load mode from straight (Figure 6a) and acutely bent crystals (Figures 6b and S14) obtained by indentation on their (01̅1̅) surface. The residual depths in the force–displacement curves of acutely bent crystals are significantly greater than those of straight crystals (Figure 6c,d). Pop-in events were observed due to the sudden release of stress when the elastic limits were reached during indenter tip penetration.^4^ Greater variation of the values of the Young’s modulus and hardness was observed, as expected, for the bent crystals, and they were also remarkably lower than those of the straight crystals (Figure 6e,f and Tables S8 and S9). The average values were *E* = 9.028 ± 0.214 and *H* = 0.198 ± 0.011 GPa for an acutely bent crystal, compared to *E* = 9.974 ± 0.159 and *H* = 0.216 ± 0.004 GPa for a straight crystal. These results show that the deformed crystals are generally softer, consistent with a previous report.^78^ For a bent crystal, the nanoindentation confirmed that the bent segment (*E* = 8.875 GPa and *H* = 0.202 GPa) is softer than the straight one (*E* = 10.348 GPa and *H* = 0.230 GPa) (Figure S15 and Table S10). Furthermore, we observed an inverse, nearly linear correlation of both hardness and modulus with increasing bending curvature (Figure 6h,i), which is consistent with the aforementioned melting point-curvature correlation trend. Heavily deformed crystals with great bending curvature often display low hardness and a small modulus (Figure 6g). Compared with the high hardness and large modulus weakening in the manually caused bending crystals (Tables S11 and S12), the softening in the naturally bent crystals is less pronounced. This difference in mechanical properties can be related to the distribution of defects in the naturally bent and manually bent crystals where these defects have been generated over very different time scales.

**Figure 6:**
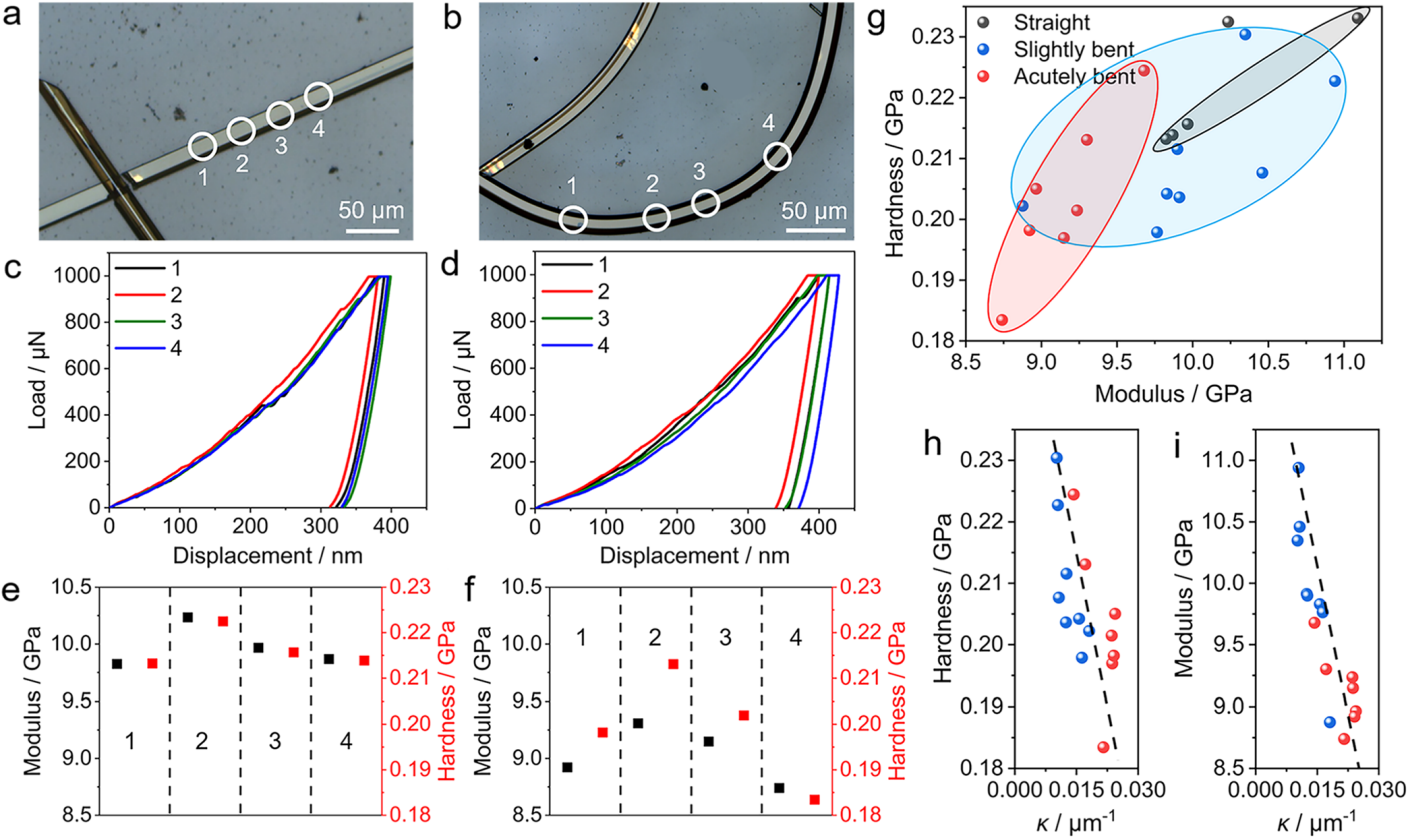
Mechanical properties of naturally bent ROY crystals. (a, b) Optical micrographs of a straight (a) and acutely bent (b) crystal with the locations labeled 1–4 that were selected for nanoindentation highlighted. (c, d) Load–displacement curves of a straight crystal recorded at locations labeled 1–4 (c) and an acutely bent crystal at locations 1–4 (d). (e, f) Young’s modulus and hardness of straight (e) and bent (f) crystals obtained by fitting the data shown in panels (c) and (d), respectively. (g) Statistics of hardness and Young’s modulus of straight, slightly bent, and acutely bent crystals. (h, i) Variation of the Young’s modulus (h) and hardness (i) with the curvature κ. The broken lines are added to guide the eye.

The observed depressions in the melting point and mechanical properties are attributed to defects accumulated in the bent crystals. In the absence of other methods for direct assessment of these effects, we turned to microfocus X-ray diffraction, which was used to analyze structural variations in straight, slightly bent, and acutely bent crystals. Thereciprocal space of the straight crystal contains well-defined diffraction spots, in line with its long-range order; however, the deformed crystals afforded diffuse diffraction spots and streaks (Figure S16), indicative of a high concentration of defects.

### Spatially Resolved Vibrational Spectroscopy

Nano-IR spectroscopy, which combines Fourier transform infrared (FTIR) and near-field scanning optical microscopy (NSOM), was used to analyze differences in both the chemistry and local structure at 10 cm^–1^ spectral resolution and a 60 nm tapping amplitude. The nano-IR spectra of a straight crystal and a bent crystal are nearly identical to those recorded from powders (Figures S17 and S18) and provide additional evidence for the phase identity of the sample. However, some discrepancies in peak shifting and intensities were observed and are attributed to method-specific factors. Conventional FTIR spectroscopy detects the direct attenuation of infrared light intensity as it passes through or is reflected by the powder sample. In contrast, the s-SNOM/nano-FTIR detects the amplitude and phase of the light scattered back by the metal probe after the localized near-field generated by the infrared light irradiated on the metal probe interacts with the specific crystal plane at the nanoscale regions, rather than transmitted or reflected light intensity. There is no noticeable spectral difference observed at different locations of a straight crystal (I, III; Figure 7a,c). In contrast, the nano-IR spectrum of a bent crystal displays some differences in relative peak intensities compared to the straight crystal. As shown in Figure 7b,d, there is a systematic change in the relative intensity ratio between two adjacent bands centered at 1252 cm^–1^ (band 1) and 1283 cm^–1^ (band 2) from 2.1 in location IV (convex side) to 1.6 in location III (concave side) (Table S12). In the straight crystals, the relative band 1-to-2 intensity ratio is around 1.9. Similar trends were also observed in the relative intensity ratios between 1350 cm^–1^ and 1624 cm^–1^ (Table S13). These observations were further confirmed by the nano-IR spectra of different bent crystals of ROY (Figures S19 and S20 and Tables S13 and S14).

**Figure 7:**
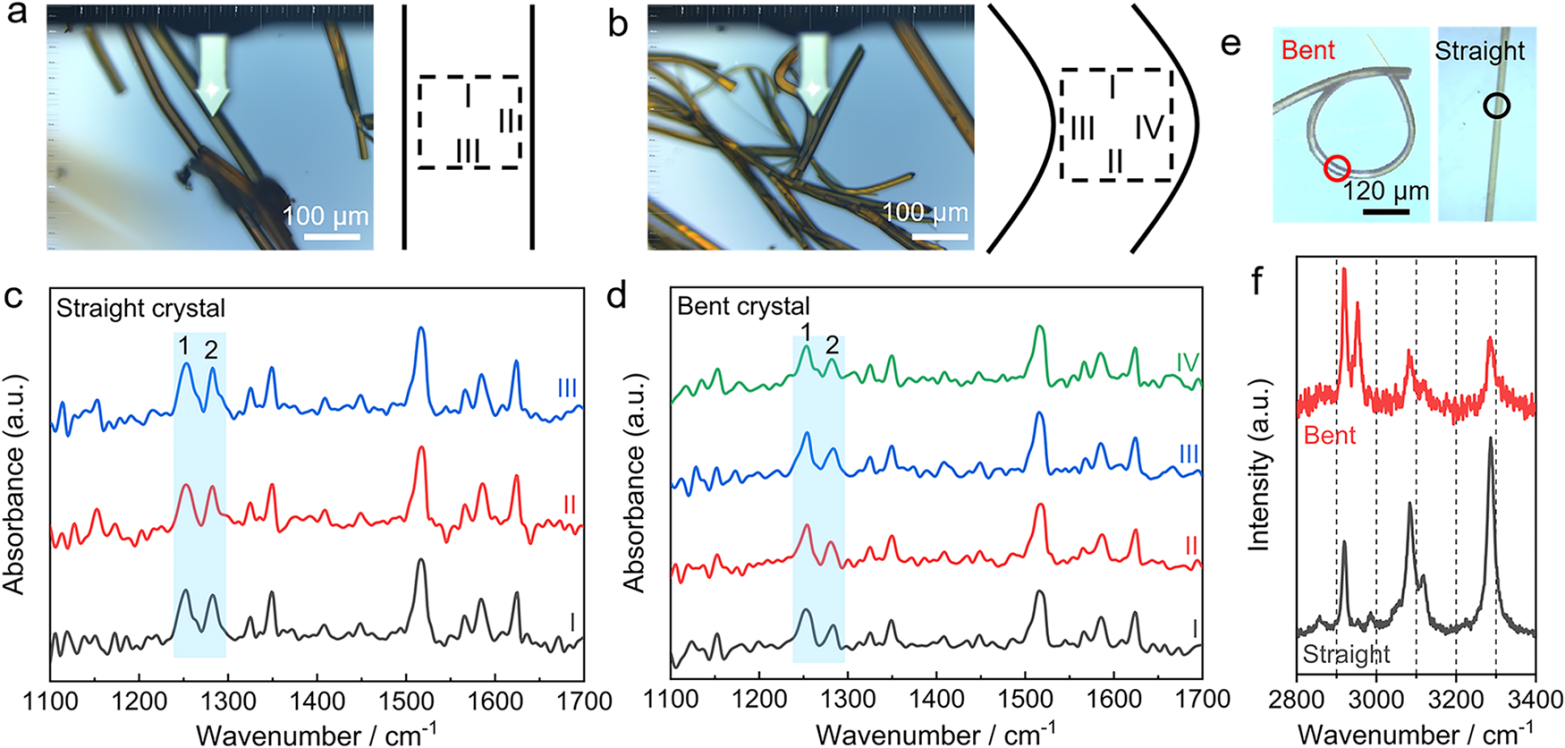
Spatially resolved vibrational spectroscopic analysis of straight and bent crystals of ROY by nano-IR and micro-Raman spectroscopy. (a, b) AFM optical micrographs of straight (a) and bent (b) crystals with schematic representations of selected locations for nano-IR measurements. (c, d) Nano-IR spectra of a straight crystal (c) at locations labeled I–III in panel (a) and a bent crystal (d) at locations I–IV in panel (b). The blue shades highlight the difference in the relative band intensity ratio 1-to-2 between the straight and bent crystals. (e) Optical micrographs of bent (left) and straight (right) crystals. (f) Micro-Raman spectra recorded on a region highlighted in panel (e) of a straight and bent crystal.

Density functional theory (DFT) calculations of the ROY molecule reveal that the band 1 at 1267 cm^–1^ is mainly associated with N=O and N–C stretching, while the band 2 at 1294 cm^–1^ is more dominantly related to N=O and C=C stretching in the aromatic ring. The bending of crystals may induce a nonuniform stress field and disruption in molecular interactions in ROY crystals, leading to a shift in the molecular orientation and configuration. We suspect that, during the growth of the bent crystals, the molecules may twist and the torsion angle θ_thio_ may change during the curving of the crystal, relaxing the strain due to accumulated energy. Indeed, DFT calculation on ROY molecules with angle θ_thio_ between −134.4° and −114.4° showed a decrease in the intensity of the band at 1267 cm^–1^ and thus reduced the band intensity ratio (Figure S21 and Table S15).

The structural perturbations were also examined on both straight and bent crystals by using micro-Raman spectroscopy. In comparison with the straight crystal, the micro-Raman spectrum of a bent crystal displays a significant peak split at 2920 cm^–1^, which is associated with the stretching vibration of the methyl C–H bonds (Figure 7e,f). The similar peak splitting upon crystal bending using manual force was also previously reported in form II of coumarin.^4^ When the molecular conformation of ROY varies with crystal bending, the local environment of the methyl C–H bond likely changes, resulting in peak splitting of this band. We also observed a remarkable change in the relative peak intensity between Raman bands at 3084 and 3287 cm^–1^. These bands are associated with the stretching vibration of the aromatic C–H and N–H bonds that involve the formation of weak hydrogen bonds C–H···O, C–H···N, and N–H···O. These interactions are expected to be affected by the molecular conformation of ROY that is reflected in the value of θ_thio_. Crystal bending often leads to compressive stress in the inner arc and tensile stress in the outer arc, and this anisotropic deformation may induce conformational variation of the flexible ROY molecules. Spatial analysis of micro-Raman spectra of cross-sections on a circular bent crystal revealed gradual change in the relative peak intensity of the bands at 1486, 1508, 1539, 1562, and 1586 cm^–1^ (Figures S22 and S23). These Raman bands are related to the stretching vibration of C=C and N=O bonds in the aromatic and thiophene rings. Collectively, these changes in the micro-Raman spectrum support the suggested conformational change of ROY molecules in the naturally bent crystals.

## Conclusions

This work reports a novel bottom-up approach to produce naturally bent ROY crystals with a circular shape and a tunable bending curvature through the microspacing sublimation technique. These naturally deformed (bent or curled) ROY crystals formed due to defects that evolve spontaneously during bottom-up crystallization. Unlike mechanically deformed plastic crystals, the deformation results in a significant depression of the melting point as large as 1.4 K. We demonstrated that this depression of the melting point of the bent or curled crystals depends upon the curvature and the crystal width; the curvature of the crystals is nearly inversely linear with a decrease in the melting point. The natural deformation of the crystals also leads to softening of the material and results in lower stiffness (as expressed by the Young’s modulus) and hardness, compared to straight crystals. The softness of the bent crystals is curvature-dependent, and both the stiffness and hardness show inverse linear correlations with the curvature. We posited that the deformation of bent ROY crystals was associated with accumulation of defects, and greater bending deformation with higher curvature corresponds to a higher density of defects. Furthermore, the high defect density in bent crystals leads to a higher chemical potential and, therefore, the first melting of bent crystals at a temperature lower than that of straight crystals. In contrast with the observation of splitting of the melting peak in DSC on mechanically plastically bent crystals,^65^ however, we observed a significant broadening of the melting peak in naturally bent crystals. This difference may be due to more evenly distributed defects in the as-grown bent crystals. Although the exact origin of defect generation in naturally bent crystals remains elusive, the spatial cross-section assay of bent segments probed by nano-IR spectroscopy shows systematic changes in the molecular conformation, which, in turn, is expected to generate internal strain. The defects induced by internal strain may raise the Gibbs energy and cause the depression of the melting point and mechanical properties. The deformed crystals exhibit deformation-related physicochemical properties, in which both melting and mechanical properties, present approximately reverse linear correlations with the bending curvature. This discovery provides a new strategy to control one of the most fundamental properties of crystalline materials simply by deformation.

## Materials and Methods

### Materials

5-Methyl-2-((2-nitrophenyl)­amino)-3-thiophenecarbonitrile (purity ≥99.5%) was purchased from Shanghai Merair Biochemical Technology Co., Ltd. and used as received.

### Hot-Stage Microscopy and Optical Microscopy

Hot-stage microscopy (HSM) was performed with a DSC600 hot-stage Linkam system with an Olympus BX-51 microscope. The samples were placed on the hot stage and were heated over the temperature range from 303.15 to 393.15 K at a constant heating rate of 5 K min^–1^, allowing for the monitoring and recording of the melting process of ROY in situ. The melting point was obtained by analyzing and recording with Perfect Decode software. The polarized optical images of the crystals were measured by a Nikon Eclipse CI-POL.

### X-ray Diffraction Analysis

Single-crystal X-ray diffraction data were collected by using Mo K_α_ radiation (λ = 0.71073 Å) or Cu K_α_ radiation (λ = 1.54184 Å) at 110 K on an Agilent-Rigaku Super Nova diffractometer with a CCD detector system. Well-grown single crystals were mounted on the loop for data collection. Data integration and reduction were carried out using the CrysAlisPro software.^84^ The intensities were corrected for Lorentz and polarization effects, and an empirical absorption correction was applied. The structures were solved by SHELXT^85^ and then refined with SHELXL.^86^ All non-hydrogen atoms were refined anisotropically, and the hydrogen atoms were refined without applying any restraints.

Powder X-ray diffraction data of ROY samples obtained by the microspacing sublimation were collected on a Rigaku D/MAX 2500 X-ray diffractometer using Cu K_α_ radiation at 40 kV and 200 mA. The samples were scanned over the 2θ range of 5–50° with a scanning rate of 8° min^–1^. Face indexing of needle–shaped ON polymorph crystals was done on a Bruker D8 Venture diffractometer (Bruker AXS Inc., Madison, WI) equipped with a Bruker PHOTON-II CMOS detector. The microfocus X-ray diffraction of ON polymorph crystals with three degrees of natural curvature was performed by using the same instrument. It should be noted that further structural analysis was not possible due to a decrease in the diffraction quality caused by the bending or curling.

### Differential Scanning Calorimetry

Differential scanning calorimetry (DSC) analysis was performed on a Mettler Toledo DSC 1/500 differential scanning calorimeter equipped with STARe software. The bent crystals growing on the glass were separated by cutting the glass and then placed in perforated aluminum crucibles, and the heating rate was set as 10 K min^–1^ in a temperature range between 298.15 and 423.15 K under a nitrogen atmosphere. Similarly, to avoid error from the glass slice in its melting point measurement, the straight crystals were placed on the glass slice and then put into a standard aluminum crucible for the DSC test, and the other test conditions were the same as those for the bent crystals. The calibration of the instrument using indium and zinc was performed to check the measurement accuracy of temperature and enthalpy before measurement.^87^

### Nanoindentation

Nanoindentation determination tests were carried out on the Bruker Hysitron TI 980 with a Berkovich tip.^88^ The crystal face was indented to a peak load of 1000 μN with a loading–unloading rate of 0.1 mN s^–1^. The resolution of load and displacement was 1 nN and 10 nm, respectively. The indenter tip had a nominal radius of about 30 nm with the pyramidal faces forming an angle of 65.27° with the vertical axis. *P*–*h* curves were analyzed by using the standard Oliver–Pharr method to extract the hardness and Young’s modulus. The Poisson’s ratio was assumed to be 0.25. To eliminate the potential contribution from the glass substrate, we performed the experiment with the glass substrate placed in the reference pan. No significant changes were observed compared to the experiments performed without the glass substrate.

### Nano-Infrared Spectroscopic Analysis

Nano-FTIR was conducted with a commercial s-SNOM/nano-FTIR setup (NeaSNOM; Neaspec GmbH). For nano-FTIR spectra, a broadband infrared laser continuum with an average output power of ∼600 μW was used. The AFM was operated in ∼250 kHz tapping mode with metallic tips (Arrow PtIr; NanoWorld AG). All nano-FTIR spectra were recorded with a 10 cm^–1^ spectral resolution and a 60 nm tapping amplitude.

### Mueller Matrix Microscopy

Mueller matrix microscopy (MMM) images were recorded by using a home-built Mueller matrix microscope, as reported in the previous work.^77,89^ It is customized with polarization state modulators based on dual-rotating waveplates. By rotating a pair of quarter waveplates, concerning two stationary crossed linear polarizers, the polarization states of light before and after the propagation of the sample are collected by a gray scale camera. All of the data are displayed as a 4 × 4 array of pseudocolor images, each representing one element of the Mueller matrix.

### Micro-Raman Spectroscopy

Raman spectra of straight and bent crystals adhered on the glass sheet were collected on a micro-Raman microscope (DXR, Thermo Fisher Scientific) equipped with a 633 nm excitation laser operating at 5 mW and a 50 μm slit. Raman spectra were recorded over a spectral range from 100–4000 cm^–1^ at a resolution of 2 cm^–1^.

### Computational Details

Infrared spectra of ROY molecules with distinct torsion angles θ_thio_ were simulated with the density functional theory (DFT)^90–93^ by using Gaussian 16 (version C.01)^94^ using the B3LYP density functional with the 6-311G­(d,p) basis set, and the molecule structure was retrieved from the Cambridge Structural Database (CSD: QAXMEH).^74^ The calculated vibrational frequencies were scaled by the factor 0.9729 to match the experimental results.^95^

## Supplementary Material

















## Notes

Movie S1. Three-point bending test of straight ROY crystal samples prepared by sublimation crystallization on the (01̅1̅) crystal plane, showing good flexibility and elastic deformation of the ON crystal form. (MP4)
Movie S2. Three-point bending test of straight ROY crystal samples prepared by sublimation crystallization on the (01̅1) crystal plane, showing good flexibility and elastic deformation of the ON crystal form. (MP4)
Movie S3. Hot-stage microscopy showing melting behavior of the ROY acutely bent crystal sample (ON form) heated over a temperature range of 386.05–393.15 K. (MP4)
Movie S4. Hot-stage microscopy showing melting behavior of the ROY curled crystal sample (ON form) heated over a temperature range of 385.65–391.15 K. (MP4)
Movie S5. Hot-stage microscopy showing melting behavior of another ROY acutely bent crystal sample (ON form) heated over a temperature range of 385.85–391.15 K. (MP4)
Movie S6. Hot-stage microscopy showing melting behavior of the ROY slightly bent crystal sample (ON form) heated over a temperature range of 387.15–392.46 K. (MP4)
Movie S7. Hot-stage microscopy showing melting behavior of the ROY straight crystal sample (ON form) heated over a temperature range of 390.65–393.25 K. (MP4)
Powder X-ray diffraction pattern; crystallographic data; observation of bent or curled crystal formation from the melt phase; |LB| and |LD| images of ROY crystals; detailed Young’s modulus and hardness data of ROY crystals; *t* test analysis; microfocus X-ray diffraction; nanoinfrared spectra of crystals; and DFT calculation results. (PDF)
