# Supplementary material for: Depression of the Melting Point in Naturally Grown Circular Crystals
Source: JACS Au. 2026 Feb 6;6(2):1036–47. doi: 10.1021/jacsau.5c01455 (PMC12933368; doi:10.1021/jacsau.5c01455)
Supplement: Supplementary file 8 [file au5c01455_si_008.pdf]

Depression of the Melting Point in Naturally Grown Circular Crystals

Shengzhe Jia[1,‡], Xin Su[1,‡], Yonghui Wang[2,‡], Jiaqiang Liu[3], Ejaz Ahmed[4], Liang Li[4,5], Weiwei Tang*[,1], Panče Naumov*[,4,6,7,8], Xiaoyan Cui*[,2], and Junbo Gong*[,1]

[1]*School of Chemical Engineering and Technology, State Key Laboratory of Chemical Engineering and Low-Carbon Technology, The Co-Innovation Center of Chemistry and Chemical Engineering of Tianjin, Tianjin University, Tianjin 300072, People's Republic of China*

[2]*Department of Chemistry, School of Chemistry and Molecular Engineering, East China Normal University, 500 Dongchuan Road, Shanghai, 200241, People's Republic of China*

[3]*College of Chemistry, Nankai University, Tianjin 300071, China*

[4]*Smart Materials Lab, New York University Abu Dhabi, P.O. Box 129188, Abu Dhabi, UAE*

[5]*Novel Materials Development Lab, Sorbonne University Abu Dhabi, PO Box 38044, Abu Dhabi, UAE*

[6]*Center for Smart Engineering Materials, New York University Abu Dhabi, PO Box 129188, Abu Dhabi, UAE*

[7]*Research Center for Environment and Materials, Macedonian Academy of Sciences and Arts, Bul. Krste Misirkov 2, MK–1000 Skopje, Macedonia*

[8]*Department of Chemistry, New York University, 100 Washington Square East, 10003, New York, USA*

‡ These authors contributed equally.

*Corresponding authors: wwtang@tju.edu.cn (W.T.); pance.naumov@nyu.edu (P.N.); xycui@chem.ecnu.edu.cn (X.C.); junbo_gong@tju.edu.cn (J.G)

This PDF file includes:

Supplementary Figures S1 to S20
Supplementary Tables S1 to S13
Captions for Supplementary Movies S1 to S7
Supplementary References

Other supplementary materials for this manuscript include the following:

Supplementary Movies S1 to S7

Supplementary Figures

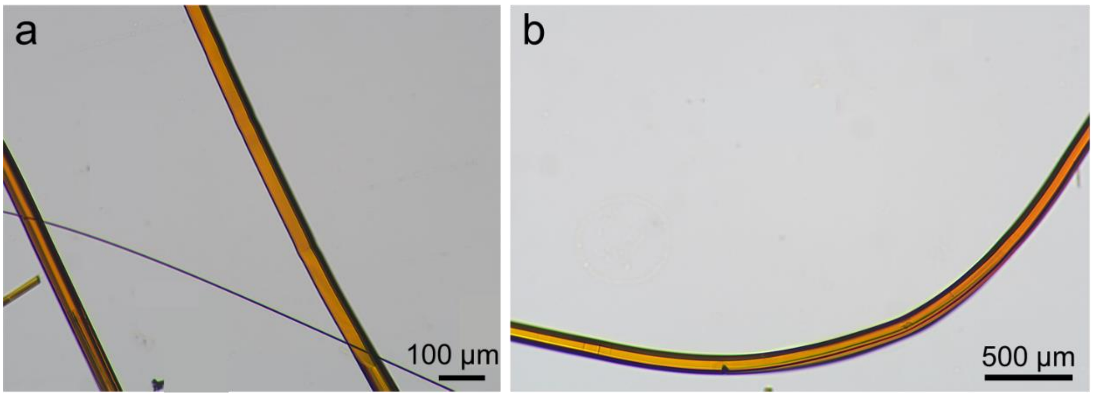

Figure S1. Polarized optical images of ROY crystals, illustrating both straight (a) and bent (b) shapes.

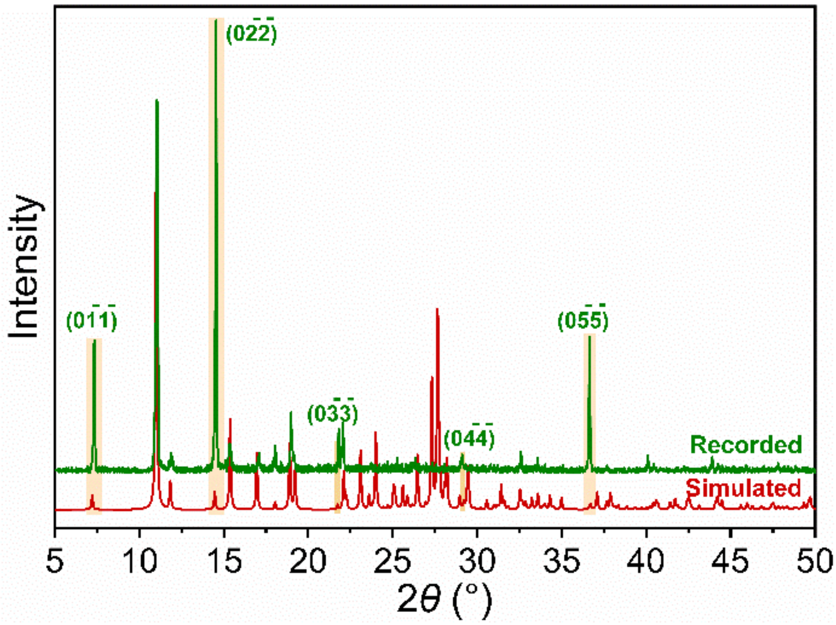

Figure S2. Comparison of recorded X-ray powder diffraction pattern of ROY crystals prepared by microspacing sublimation with the pattern simulated for the ON form. The regions highlighted with yellow background indicate the preferred orientation of the crystal of the ON form produced by sublimation.

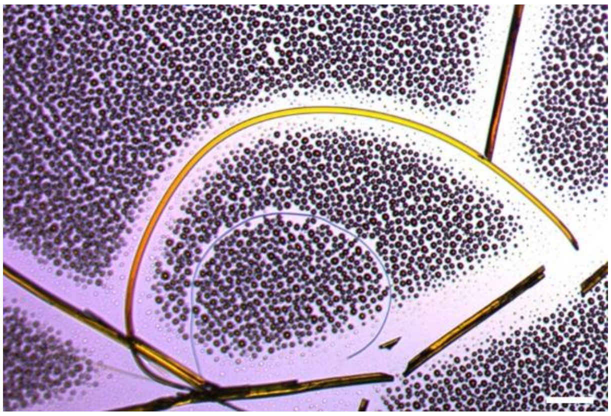

Figure S3. Optical images of ROY crystals prepared by the microspacing sublimation show the formation of curled crystals from the melt. Scale bar: 100 μm.

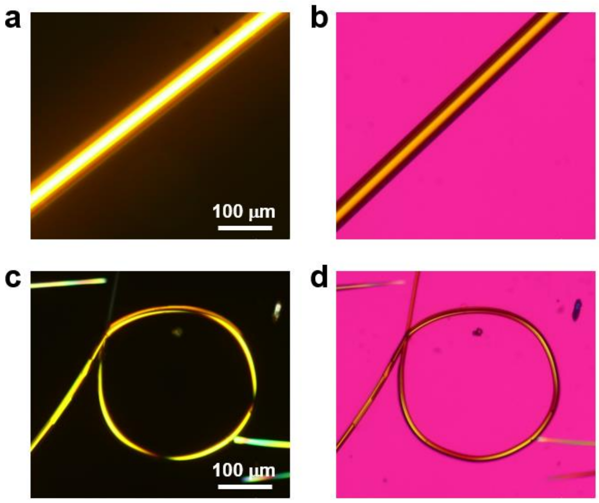

Figure S4. Polarized micrographs for (a, b) straight and (c, d) circular crystals under crossed polarizers (a, c) and recorded by using a retarder (b, d).

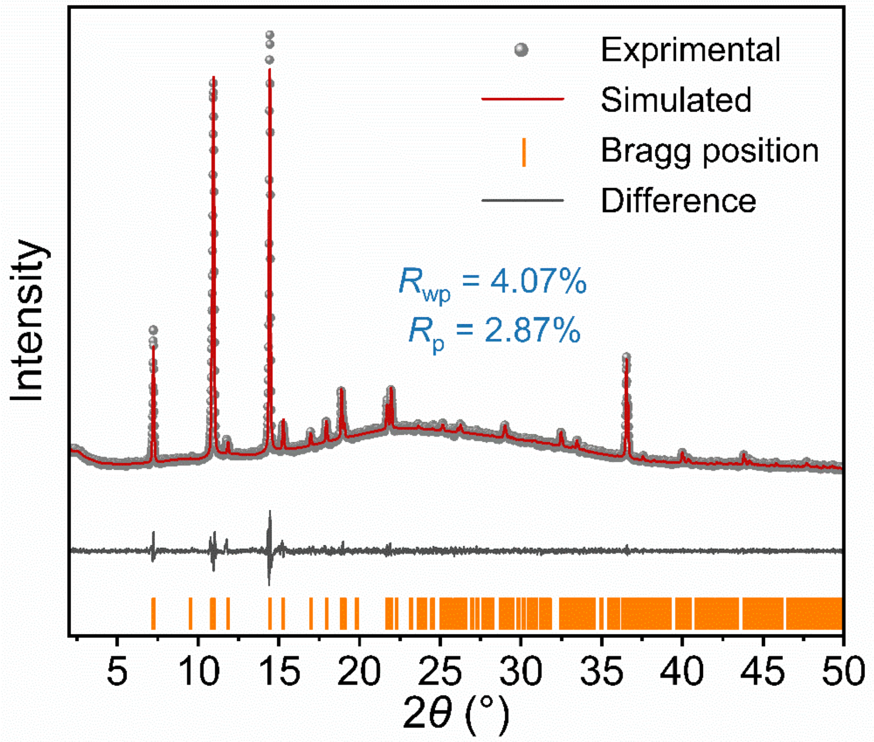

Figure S5. The Rietveld refinement of collected PXRD data.

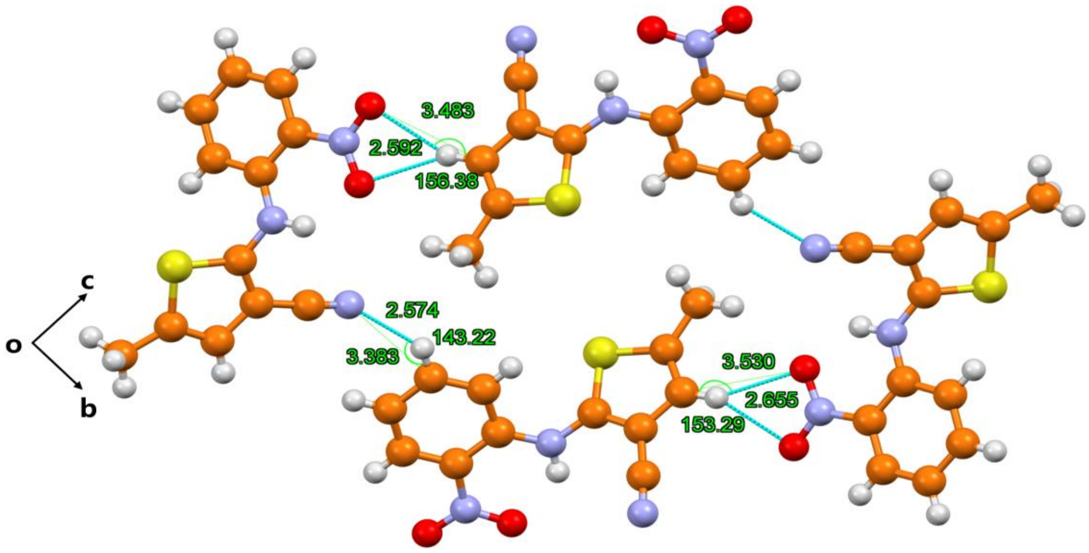

Figure S6. Basic parameters for the intermolecular interactions in the crystal of the ON form shown in the direction of the crystallographic *a* axis.

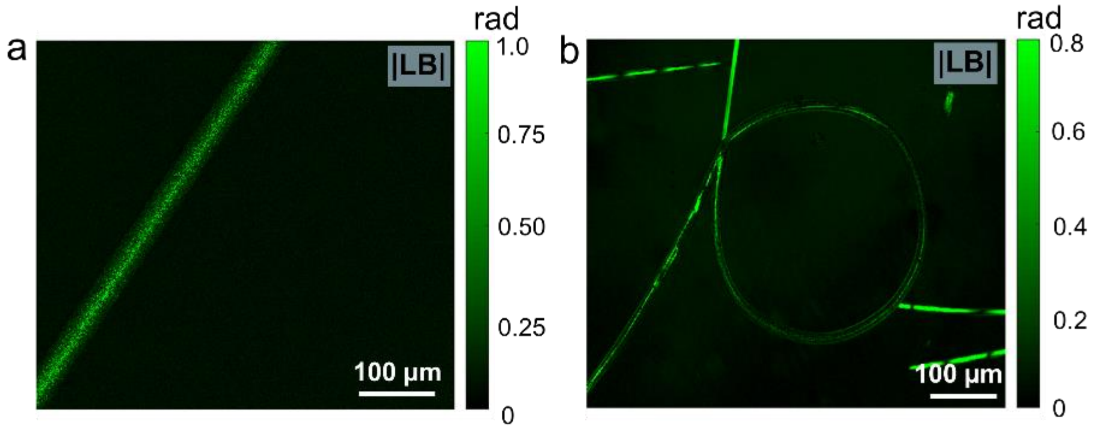

Figure S7. |LB| angle mapping of straight (a) and curled (b) crystal of ROY.

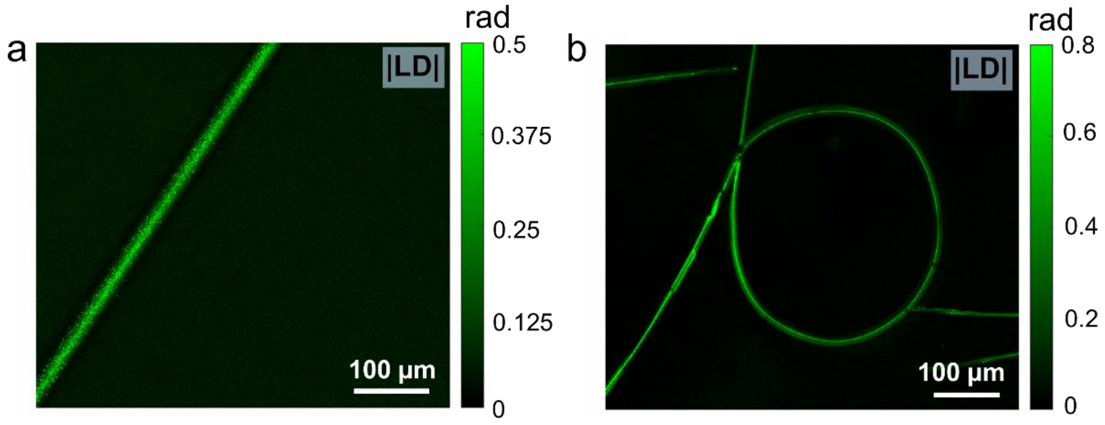

Figure S8. |LD| angle mapping of straight (a) and curled (b) crystal of ROY.

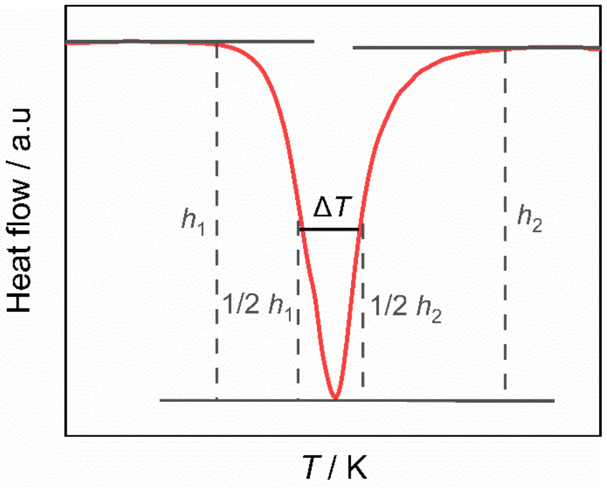

Figure S9. Schematic illustration of the definition and calculation of the half-peak width ΔT.

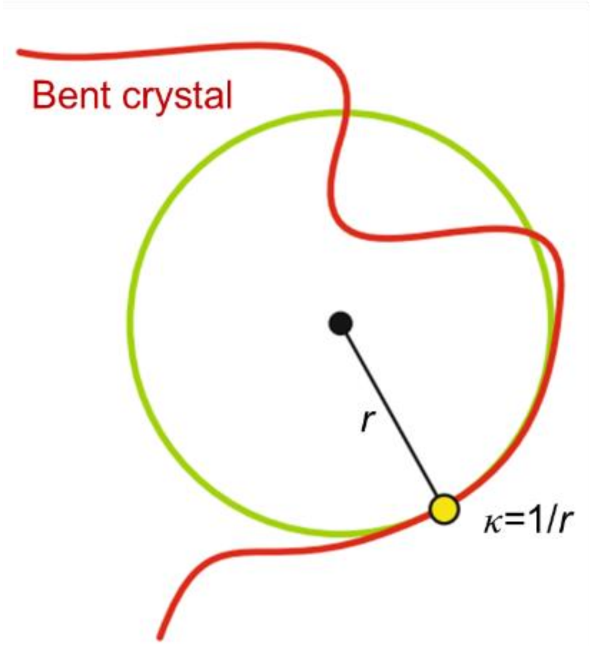

Figure S10. Schematic representation of calculation of the curvature at a given point of bent or curled crystal.

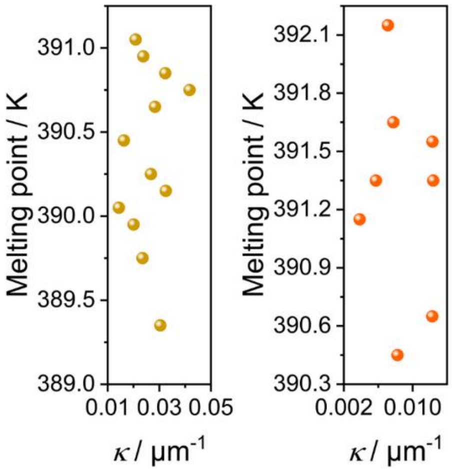

Figure S11. Categorization of the melting point with the curvature (κ) based on the crystal width: 1–2 μm (left) and 8–16 μm (right).

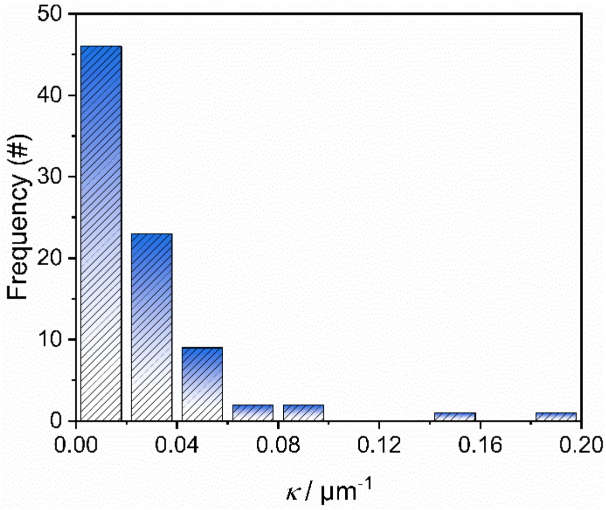

Figure S12. Curvature distribution of naturally curved ROY ON crystals.

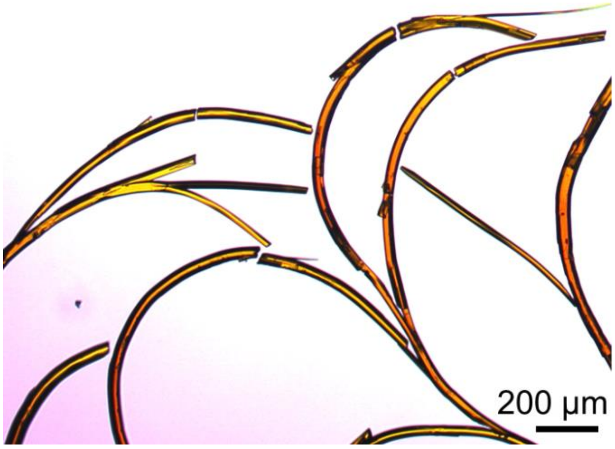

Figure S13. Optical images of bent ROY crystals with defects on their surfaces.

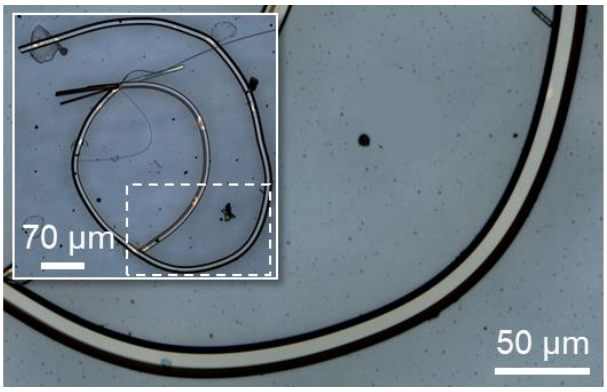

Figure S14. Optical micrograph displaying a selected region of an acutely bent ROY crystal used for nanoindentation. The inset provides a larger view of the crystal at a lower magnification. The portion of the crystal outlined with a white dashed line is zoomed in and shown in the larger image.

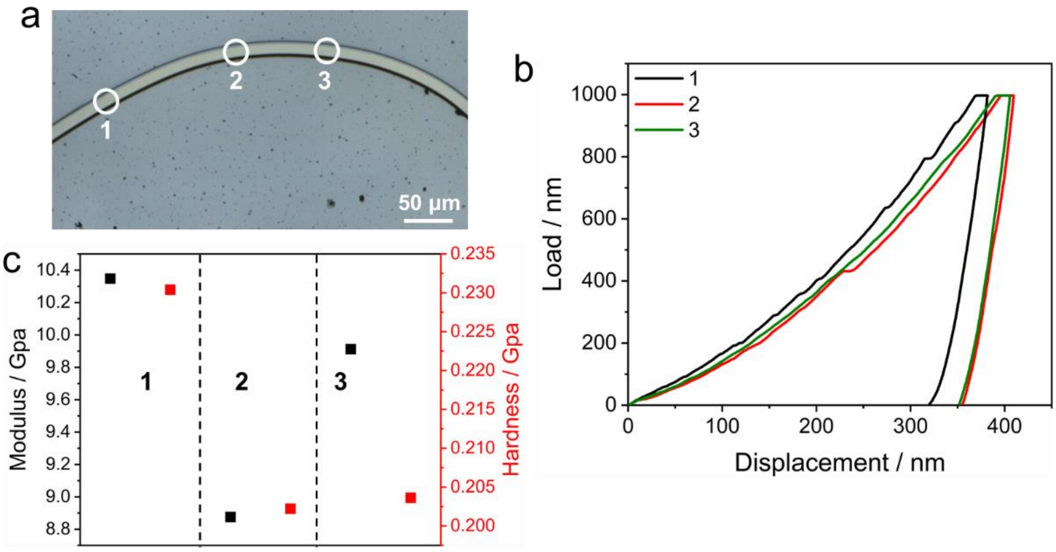

Figure S15. Mechanical properties of a slightly bent crystal. (a) Optical micrographs of a slightly bent crystal with locations labeled 1–3 that were selected for nanoindentation highlighted. (b) Load-displacement curves recorded at locations 1–3 on the crystal shown in panel a. (c) Young's modulus and hardness of a slightly bent crystal obtained by fitting the data shown in panel b.

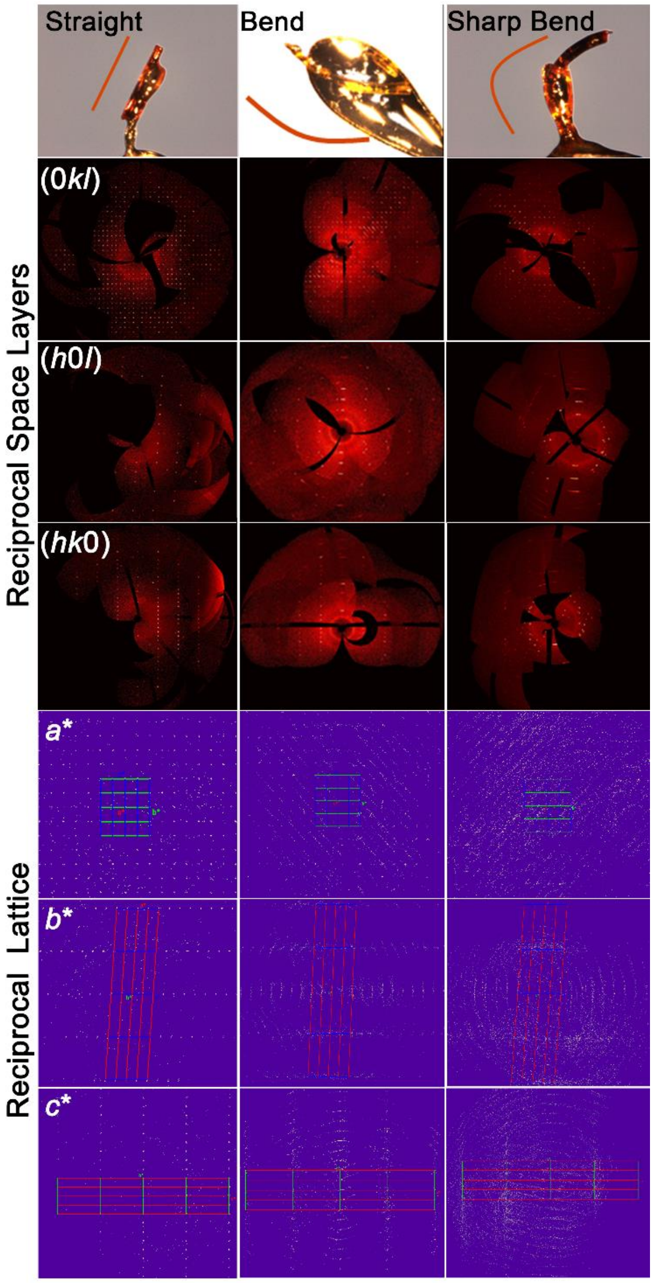

Figure S16. Structures of straight and deformed crystals analyzed by microfocus X-ray diffraction. Microphotographs (top), reciprocal space layers (middle), and reciprocal lattice reconstructed from the crystallographic data (bottom) are shown.

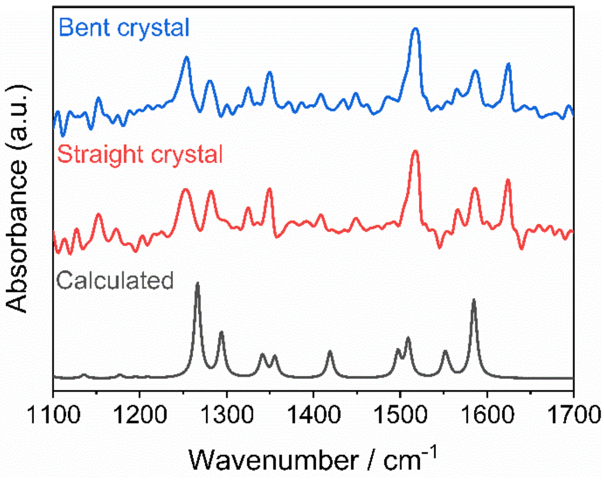

Figure S17. Comparison of the calculated IR spectrum of ROY molecule at the B3LYP/6-311G (d,p) level using DFT,[S2] with nano-FTIR spectra of straight and bent crystals of ROY.

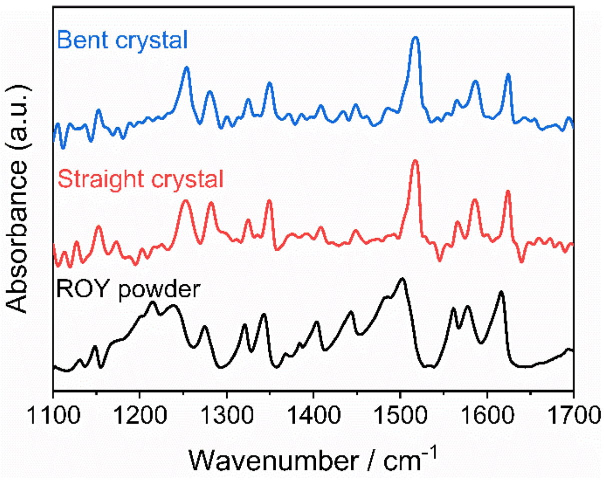

Figure S18. Comparison of nano-FTIR spectra of the straight and bent crystals of ROY with FTIR spectrum measured from bulk ROY crystal powder.

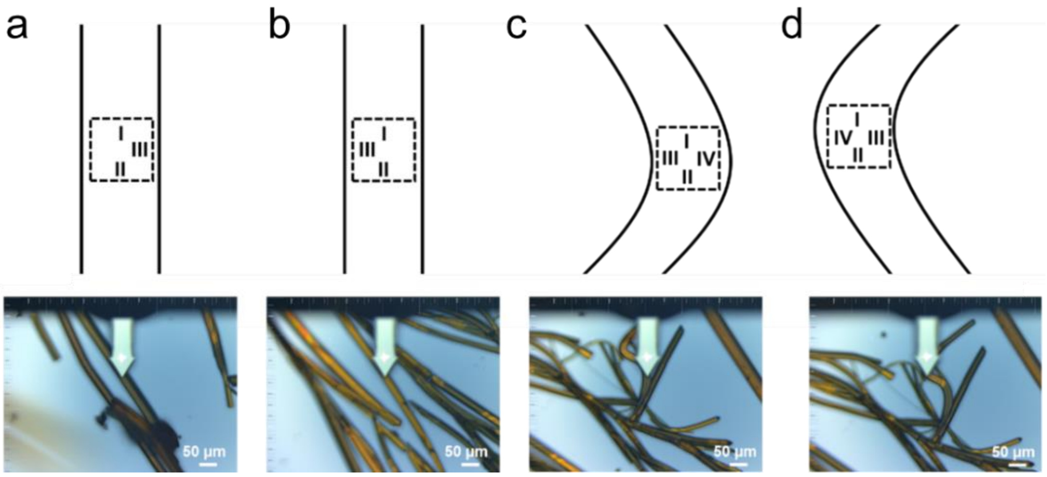

Figure S19. Optical micrographs taken by the aid of atomic force microscope of additional straight (a, b) and bent (c, d) crystals. The sketches on the top show the locations where the nano-IR spectra were recorded.

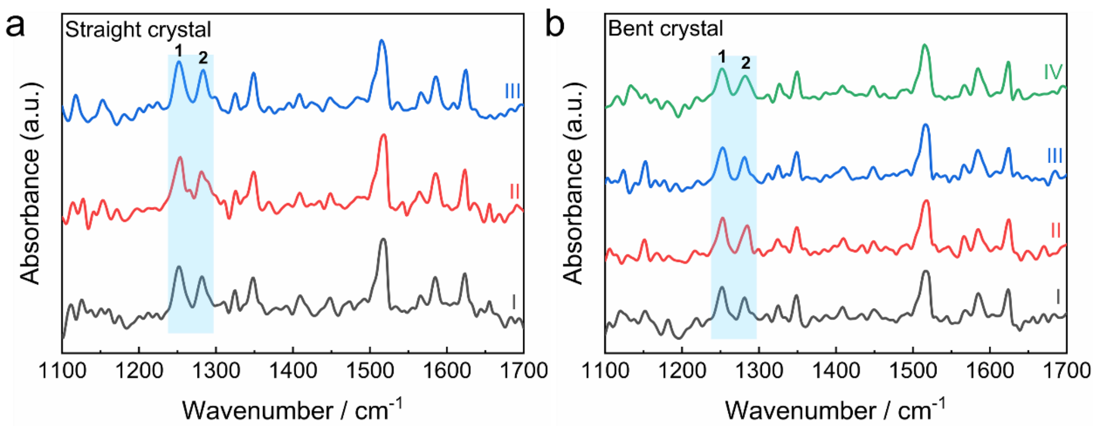

Figure S20. Nano-IR spectra recorded at the locations of the straight crystal labeled I–III in Figure S16a (a) and at the locations labeled I–IV of the bent crystal in Figure S16c (b). The blue-shaded background highlights the difference in the relative intensity ratio of the bands 1 and 2 between the straight and the bent crystal.

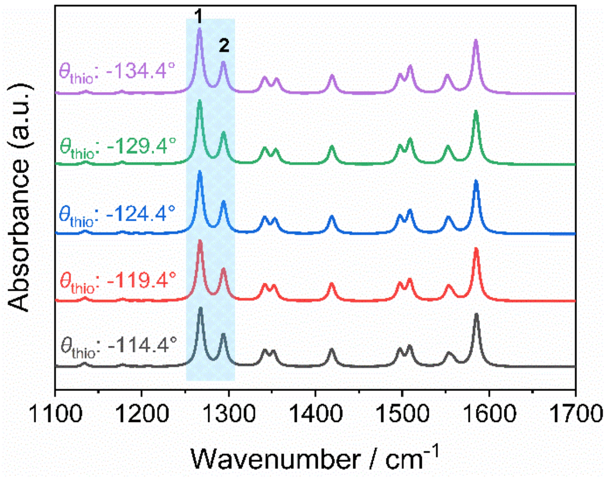

Figure S21. Theoretical DFT IR spectra of ROY molecules with varying torsion angles θ_{thio} calculated at the B3LYP/6-311G(d,p) level.[S3] The relative band intensity ratio decreases with increasing torsion angle θ_{thio}.

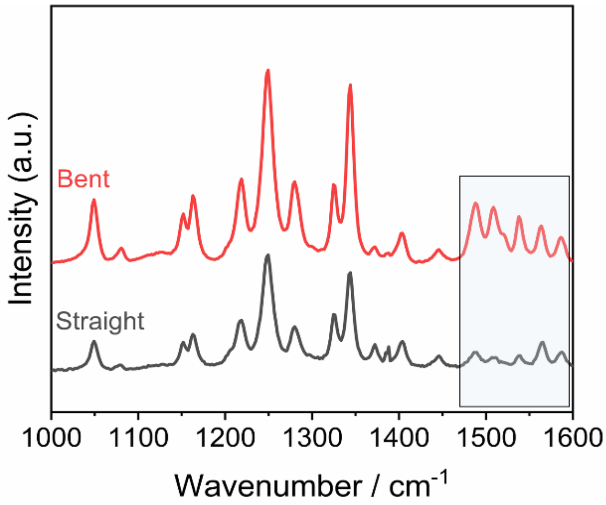

Figure S22. Experimental micro-Raman spectra of straight and bent crystals of ROY in the wavenumber range 1000–1600 cm^{-1}. The box highlights region of pronounced spectral difference between the straight and bent crystals.

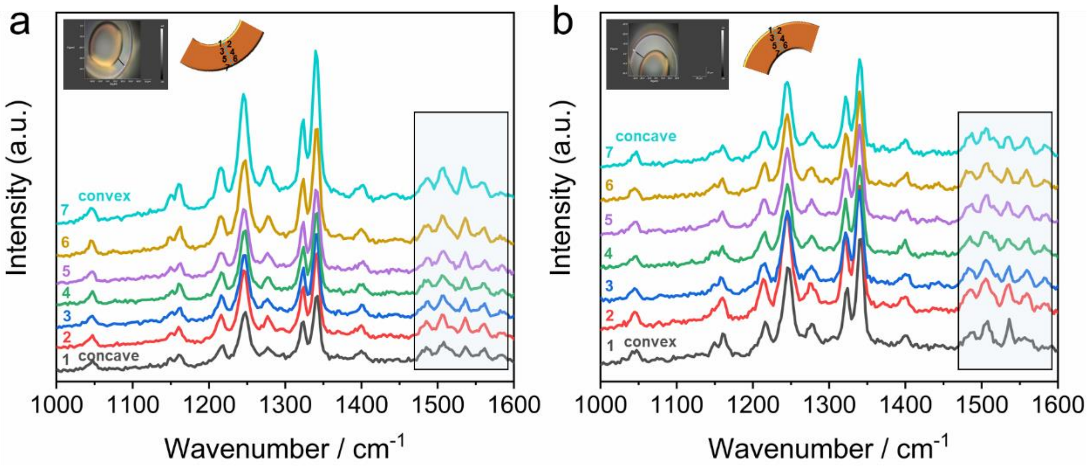

Figure S23. Spatially resolved vibrational spectroscopic analysis of a bent ROY crystal by micro-Raman spectroscopy. The non-polarized Raman spectra were recorded from the regions (highlighted in the insets) from the concave side (position 1) to convex side (position 7) of a bent segment (a), and from the convex side (position 1) to the concave side (position 7) on a segement away from the kink (b).

Supplementary Tables

Table S1. Crystallographic data and refinement details of the ON crystal form of ROY.

Basic parameters	ROY
Temperature / K	110
Polymorph	ON
Formula	$C_{12}H_9N_3O_2S$
Formula weight	259.28
Crystal system	Monoclinic
Space group	$P2_1/c$
a / Å	3.8552(1)
b / Å	18.4685(4)
c / Å	16.3436(3)
α / °	90
β / °	92.583(2)
γ / °	90
Volume /Å^3	1162.48(4)
Z	4
Density / (g cm^{-3})	3.7
F_{000}	142
h_{min}, h_{max}	-4, 4
k_{min}, k_{max}	-22, 22
l_{min}, l_{max}	-20, 20
No. of measured reflections	8436
No. of unique reflections	5727
No. of reflections used	5346
R_{all}, R_{obs}	0.0352, 0.0352
$wR_{2,all}$, $wR_{2,obs}$	0.0949, 0.0949
$\Delta\rho_{min,max}$ / (e Å^{-3})	-0.3, 0.3
GooF	1.083

Table S2. Crystallographic data for the ON form of ROY solved by single-crystal analysis and PXRD refinement in this work.

Basic parameters	Single-crystal analysis in this work	PXRD Refinement
Polymorph	ON	ON
Formula	$C_{12}H_9N_3O_2S$	$C_{12}H_9N_3O_2S$
Formula weight	259.28	259.28
Crystal system	Monoclinic	Monoclinic
Space group	$P2_1/c$	$P2_1/c$
a / Å	3.8552(1)	3.8383
b / Å	18.4685(4)	18.6127
c / Å	16.3436(3)	16.3305
α / °	90	90
β / °	92.583(2)	92.6125
γ / °	90	90
Volume /Å^3	1162.48(4)	1165.46

Table S3. Statistics of melting point data measured from at least 8 individual straight crystals.

Sample number	Melting point / K
1	390.63
2	390.62
3	390.39
4	390.21
5	390.22
6	389.99
7	390.06
8	390.34
Average value / K	390.31
Standard deviation / K	0.22

Table S4. Statistics of melting point data measured from at least 8 individual bent crystals.

Sample number	Melting point / K
1	388.33
2	388.84
3	389.43
4	388.47
5	388.99
6	388.48
7	389.12
8	389.53
Average value / K	388.90
Standard deviation / K	0.42

Table S5. t-Test performed on the melting point data of straight and bent crystals (Tables S2 and S3). The probability was found to be lower than 0.001, which indicates that there is a significant difference in the melting point of the bent and unbent crystals.

t	*df*	*P*	Cohen's d
9.857	7	< 0.001	3.486

Table S6. Statistics of melting point data measured from at least 8 individual bent crystals with different crystal widths.

Crystal width / μm	1–2	2–2.5	2.5–3	3–4	4–6	6–8	8–16
	389.35	389.95	390.45	390.85	390.75	391.05	391.35
	390.05	391.05	389.85	390.25	389.45	391.35	390.65
	390.85	391.05	391.05	390.65	389.95	390.95	391.15
	389.95	390.35	389.15	390.75	391.35	391.25	390.45
	390.15	390.65	390.45	389.85	389.45	390.75	391.65
	390.65	390.35	390.55	390.85	390.75	391.35	392.15
	390.75	389.85	390.25	390.75	390.15	390.85	391.35
Melting point / K	391.05	390.25	390.95	390.45	390.85	390.95	391.55
	389.75	390.05	389.65	390.75	390.95	390.35	
	390.45	390.35		390.85	389.75	390.15	
	390.25	390.45		390.65	390.45	391.15	
	390.95	390.35		390.35	388.65	390.05	
		390.45		390.05	391.25	391.65	
				390.55	390.55	390.95	
Standard deviation / K	0.50	0.35	0.58	0.31	0.77	0.46	0.51

Table S7. Linear fitting correlation of the melting point of curved crystals with the bending curvature.

Crystal width	Fitting correlation
2–2.5 μm	$T_m = 390.74 - 5.259\times\kappa$, $R^2 = 0.5722$
2.5–3 μm	$T_m = 390.91 - 15.582\times\kappa$, $R^2 = 0.6762$
3–4 μm	$T_m = 390.89 - 16.976\times\kappa$, $R^2 = 0.6603$
4–6 μm	$T_m = 391.25 - 70.750\times\kappa$, $R^2 = 0.6582$
6–8 μm	$T_m = 391.87 - 79.498\times\kappa$, $R^2 = 0.8610$

Table S8. Statistics of Young's modulus and hardness of a straight crystal at locations from 1 to 4 (See Figure 6a).

Location	Modulus / GPa	Hardness / GPa
1	9.8247	0.2132
2	10.2346	0.2225
3	9.9670	0.2157
4	9.8680	0.2139
Average value / GPa	9.974	0.216
Standard deviation / GPa	0.159	0.004

Table S9. Statistics of Young's modulus and hardness of an acutely bent crystal at locations from 1 to 4 (See Figure 6b).

Location	Hardness / GPa	Modulus / GPa
1	8.9212	0.1982
2	9.3009	0.2131
3	9.1488	0.1970
4	8.7396	0.1834
Average value / GPa	9.028	0.198
Standard deviation	0.214	0.011

Table S10. Statistics of Young's modulus and hardness of a slightly bent crystal at locations from 1 to 3 (See Figure S12a).

Location	Hardness / GPa	Modulus / GPa
1	10.3479	0.2304
2	8.8754	0.2022
3	9.9125	0.2036
Average value / GPa	9.712	0.212
Standard deviation	0.618	0.013

Table S11. Comparison of the hardness between mechanically bent and naturally grown bending crystals.

Crystal	Hardness (straight) / GPa	Hardness (bent) / GPa	Reduction percentage / %
Hexachlorobenzene[S4]	0.084	0.009	89.29
1,4-dibromobenzene[S1]	0.061	0.037	39.34
ROY (this work)	0.216	0.198	8.33

Table S12. Comparison of the Young's modulus between mechanically bent and naturally grown bending crystals.

Crystal	Modulus (straight) / GPa	Modulus (bent) / GPa	Reduction percentage / %
Hexachlorobenzene[S4]	5.30	0.272	94.87
1,4-dibromobenzene[S1]	4.309	3.196	25.83
ROY (this work)	9.974	9.028	9.49

Table S13. Variation of the relative band intensity ratio between band 1 (1252 cm^{-1}) and band 2 (1283 cm^{-1}) in the nano-FTIR spectra. The parameter displays a systematic change from the convex side to the concave side.

Sample	Position	Average band 1-to-2 ratio
Straight crystal 1		1.88
Straight crystal 2		1.89
Bent crystal 1	Location IV	2.12
	Locations I, II	1.82
	Location III	1.62
Bent crystal 2	Location IV	2.05
	Locations I, II	1.75
	Location III	1.61

Table S14. Variation of the relative intensity ratio between band 3 (1350 cm^{-1}) and band 4 (1624 cm^{-1}) in the nano-FTIR spectra. The parameter displays a systematic change from the convex side to the concave side.

Sample	Position	Average band 3-to-4 ratio
Straight crystal 1		0.94
Straight crystal 2		0.96
Bent crystal 1	Location IV	1.03
	Locations I, II	0.76
	Location III	0.68
Bent crystal 2	Location IV	1.01
	Locations I, II	0.84
	Location III	0.80

Table S15. The variation of the relative band intensity ratio 1-to-2 in the calculated IR spectra of ROY molecules at different torsion angles θ_{thio}.

θ_{thio}	Band 1 (area)	Band 2 (area)	Band 1 / Band 2
-114.4°	10896.3	5008.5	2.18
-119.4°	11317.6	4986.8	2.27
-124.4°	11776.2	4982.8	2.36
-129.4°	12072.8	4962.0	2.43
-134.4°	12381.2	4938.6	2.51

Legends for the Supplementary Movies

Movie S1. Three-point bending of straight ROY crystal samples prepared by sublimation crystallization, bent on their $(0\bar{1}\bar{1})$ facet, showing favorable flexibility and elastic deformation. Snapshots of the movie are shown in Fig. 2d.

Movie S2. Three-point bending of straight ROY crystal samples prepared by sublimation crystallization, bent on their $(0\bar{1}1)$ facet, showing favorable flexibility and elastic deformation. Snapshots of the movie are shown in Fig. 2c.

Movie S3. Hot-stage microscopic observation of the melting of an acutely bent ROY crystal (ON form) heated over the temperature range from 386.05 to 393.15 K. Shapshots of the movie are shown in Fig. 4a.

Movie S4. Hot-stage microscopic observation of the melting of a curled ROY crystal (ON form) heated over the temperature range from 385.65 to 391.15 K.

Movie S5. Hot-stage microscopic observation of the melting of a second acutely bent ROY crystal (ON form) heated over the temperature range from 385.85 to 391.15 K.

Movie S6. Hot-stage microscopic observation of the melting of a slightly bent ROY crystal (ON form) heated over the temperature range from 387.15 to 392.46 K

Movie S7. Hot-stage microscopic observation of the melting of a straight ROY crystal (ON form) heated over the temperature range from 390.65 to 393.25 K.

Supplementary References

(S1) Ahmed, E.; Karothu, D. P.; Pejov, L.; Commins, P.; Hu, Q.; Naumov, P. From Mechanical Effects to Mechanochemistry: Softening and Depression of the Melting Point of Deformed Plastic Crystals. *J. Am. Chem. Soc.* **2020**, *142*, 11219−11231.

(S2) Oomens, J.; Morton, T. H. The Cationic $C{=}F^+$ Stretching Vibration in the Gas Phase. *Angew. Chem. Int. Ed.* **2008**, *47*, 2106–2108.

(S3) Frisch, M. J.; Trucks, G. W.; Schlegel, H. B.; Scuseria, G. E.; Robb, M. A.; Cheeseman, J. R.; Scalmani, G.; Barone, V.; Petersson, G. A.; Nakatsuji, H.; Li, X.; Caricato, M.; Marenich, A. V.; Bloino, J.; Janesko, B. G.; Gomperts, R.; Mennucci, B.; Hratchian, H. P.; Ortiz, J. V.; Izmaylov, A. F.; Sonnenberg, J. L.; Williams; Ding, F.; Lipparini, F.; Egidi, F.; Goings, J.; Peng, B.; Petrone, A.; Henderson, T.; Ranasinghe, D.; Zakrzewski, V. G.; Gao, J.; Rega, N.; Zheng, G.; Liang, W.; Hada, M.; Ehara, M.; Toyota, K.; Fukuda, R.; Hasegawa, J.; Ishida, M.; Nakajima, T.; Honda, Y.; Kitao, O.; Nakai, H.; Vreven, T.; Throssell, K.; Montgomery Jr., J. A.; Peralta, J. E.; Ogliaro, F.; Bearpark, M. J.; Heyd, J. J.; Brothers, E. N.; Kudin, K. N.; Staroverov, V. N.; Keith, T. A.; Kobayashi, R.; Normand, J.; Raghavachari, K.; Rendell, A. P.; Burant, J. C.; Iyengar, S. S.; Tomasi, J.; Cossi, M.; Millam, J. M.; Klene, M.; Adamo, C.; Cammi, R.; Ochterski, J. W.; Martin, R. L.; Morokuma, K.; Farkas, O.; Foresman, J. B.; Fox, D. J. Gaussian 16 Rev. C.01, Wallingford, CT, **2016**.

(S4) Panda, M.; Ghosh, S.; Yasuda, N.; Moriwaki, T.; Mukherjee, G.; Reddy, C.; Naumov, P. Spatially Resolved Analysis of Short-Range Structure Perturbations in a Plastically Bent Molecular Crystal. *Nat. Chem.* **2014**, *7*, 65−72.
